# Supplementary material for: Microbes contribute to setting the ocean carbon flux by altering the fate of sinking particulates
Source: Nat Commun. 2022 Mar 29;13:1657. doi: 10.1038/s41467-022-29297-2 (PMC8964765; doi:10.1038/s41467-022-29297-2)
Supplement: Supplementary file 1 — Supplementary Information [file 41467_2022_29297_MOESM1_ESM.pdf]

Supplementary Information for "Microbes contribute to setting the ocean carbon flux by altering the fate of sinking particulates"

Trang T. H. Nguyen[1], Emily J. Zakem[1], Ali Ebrahimi[2], Julia Schwartzman[2], Tolga Caglar[3], Kapil Amarnath[3], Uria Alcolombri[4], François J. Peaudecerf[4], Terence Hwa[3], Roman Stocker[4], Otto X. Cordero[2], Naomi M. Levine[1,(*)]

[1] Department of Biological Sciences, University of Southern California, Los Angeles, CA 91011, USA

[2] Ralph M. Parsons Laboratory for Environmental Science and Engineering, Department of Civil and Environmental Engineering, Massachusetts Institute of Technology, Cambridge, MA 02139, USA

[3] Department of Physics, University of California at San Diego, La Jolla, CA 92093, USA

[4] Institute of Environmental Engineering, Department of Civil, Environmental and Geomatic Engineering, ETH Zurich, 8093 Zurich, Switzerland

[(*)] Corresponding author: n.levine@usc.edu

Methods and model equations are presented in Supplementary Material 1. A list of model parameters and their units is presented in Supplementary Table 1. Model sensitivity tests of key assumptions and choices of parameter values are presented in Supplementary Material 2. The primary physical and biological dynamics of the model are discussed in additional detail in Supplementary Material 3. The detailed analytical calculation for the density-dependent growth on the surface of the particulate organic carbon is described in Supplementary Material 4. The measurements of bacterial growth rates for *Vibrio* sp. 1A01 across different temperatures are provided in Supplementary Table 2. The model code is deposited on GitHub (https://github.com/LevineLab/POMmodel) and citable using DOI: 10.5281/zenodo.6015020.

SUPPLEMENTARY MATERIAL 1: Methods

The model presented in this work captures key micro-scale dynamics occurring on particulate organic carbon (POC) in a manner scalable to the water column. Specifically, the model represents the consumption of POC by heterotrophic bacteria and archaea (for short we will refer to this group as either microbes or bacteria). Dynamics include in the model are the enzymatic degradation of polymer to low molecular weight organic matter (LMWOM), the density dependent growth of heterotrophic microbes, and the attachment and detachment of heterotrophic microbes to/from particles.

The model tracks each unique particle type i (defined based on radius at formation and lability) separately. This model can be coupled to a full ecosystem model such that the generation of particle type is calculated prognostically. Here, we focus on the degradation of POC below the export depth and so simply include a source term to represent particle formation. We use a default particle formation depth of 100 m but also test the sensitivity of the model results to other formation depths (Supplementary Material 2). For simplicity, we also do not allow for particle aggregation or disaggregation to occur within the water column. The results presented in the main text make the simplifying assumption that each particle type i consists of a single lability of organic matter that is colonized by a specific type of bacterial primary degrader. For the results presented in the main text, we represent particle-associated microbial diversity using 18 groups of heterotrophic microbes, defined based on their enzyme kinetics and growth rates. In reality, particles are composed of a wide range of organic compounds and consumed by a diverse community of microbes. We conduct tests in which the model is configured to represent complex particles and multiple bacterial groups. These sensitivity tests show that the degradation dynamics of the complex particles are qualitatively the same as simulations with many particles each composed of a single lability across the same spectrum as represented in the complex particles (Supplementary Material 2, Supplementary Figure 2). To reduce complexity and computational requirements, we therefore use the simpler representation of a single POC type and single degrader class per particle (total of 18 bacterial groups). It is also important to note that our bacterial groups should not be considered bacterial species. Rather, bacterial group B_i represents all species that can consume POC type i at rate β_i (eq. s1 below). For example, if particle i was composed primarily of chitin then bacterial group B_i would consist of all species with similar processing rates of chitin.

As each particle sinks through the water column, POC is consumed by the particle-associated microbial community. The change in the carbon content of the particle ($C_{poc,i}$, mmol C particle^{-1}) over time is defined as:

$$\frac{dC_{poc,i}}{dt} = -\beta_i B_i \tag{s1}$$

where β_i (mmol C_{POC} mmol C_{cell}^{-1} day^{-1}) represents the polymer degradation rate of $C_{poc,i}$ to LMWOM by bacterial group B_i (mmol C_{cell}^{-1} particle^{-1}). Specifically, β_i captures differences in the 'lability' of particles, which is a function the organic matter itself, the bacterial enzymes specific to group B_i, and the production rate of those enzymes by B_i. We assume that there is no loss of carbon during the enzymatic cleavage from POC to LMWOM such that 1 mmol C_{POC} degraded = 1 mmol C_{lmwom} produced – there is however diffusive loss of LMWOM away from the particle as described below.

The enzymatic degradation of POC results in the production of LMWOM (C_{lmwom}), similar to[1], which supports bacterial growth. We track the concentration of LMWOM at the particle surface (C_{lmwom}, mmol C m^{-3}) as:

$$\frac{dC_{lmwom,i}}{dt} = \left(\beta_i B_i - d_{loss} C_{lmwom} - \frac{\mu_i B_i}{y_{lmwom}}\right)/V \tag{s2}$$

where μ_i is the growth rate of bacteria B_i on the particle (day^{-1}) (see eq. s8), y_{lmwom} is the aerobic bacterial growth efficiency (mmol C_{cell} mmol C_{lmwom}^{-1}, here set to 0.2)[2], d_{loss} is the diffusion loss rate of LMWOM (m^3 day^{-1} particle^{-1}), and V is the small volume around the particle inhabited by particle-attached bacteria. We make the simplifying assumption that the steady-state LMWOM concentration is reached rapidly such

that equation s2 can be set equal to zero and C_{lmwom} can be solved for (main text eq. 2), see Supplementary Material 4 for this derivation. We confirm that the steady-state approximation reproduces the same results as explicitly resolving the LMWOM dynamics around a particle (Supplementary Material 2, Supplementary Figure 22).

Diffusive loss is calculated as:

$$d_{loss} = 4\pi r_i Sh_i D \quad \text{(s3)}$$

in which r_i is the particle radius (cm), D is the molecular diffusion coefficient for a chemical solute in water (here we use the coefficient for glucose, 6×10^{-6} cm^2 s^{-1})[3]. Sh_i is the Sherwood number and represents relative contribution of diffusive and advective loss, where *Sh*=1 represents pure diffusive loss and elevated *Sh* values includes significant advective loss away from the particle[4,5]. Sh_i is calculated as:

$$Sh_i = 1 + 0.619 Re_i^{0.412}\, Sc^{\frac{1}{3}} \quad \text{(s4)}$$

where Re_i is the Reynold number that represents the ratio of inertial forces to viscous forces[5,6] and is calculated as:

$$Re_i = \frac{\omega r_i}{v} \quad \text{(s5)}$$

Sc is the Schmidt number and is defined as:

$$Sc = \frac{v_{kinematic}}{D} \quad \text{(s6)}$$

where $v_{kinematic}$ is kinematic viscosity of seawater, 10^{-2} cm^2 s^{-1} [7,8].

Bacterial dynamics on each particle are defined as:

$$\frac{dB_i}{dt} = (\mu_i - L_i - m_{lin,i})B_i + E_{i,z} \quad \text{(s7)}$$

where L_i is the net bacterial detachment rate from the particle (day^{-1}), $m_{lin,i}$ is the mortality rate for bacterial group i (day^{-1}). The microbial encounter rate ($E_{i,z}$, mmol C_{cell} day^{-1} particle^{-1}) represents the rate of colonization of the particle by the free-living microbial pool of group *i* at depth *z* (eq. s11). The bacterial growth rate, μ_i (day^{-1}), is dependent on the LMWOM concentration at the particle surface and is represented with the Monod equation:

$$\mu_i = \mu_{max,i} \frac{C_{lmwom}}{C_{lmwom} + k_{m,i}} \gamma_{T,z} \quad \text{(s8)}$$

where $\mu_{max,i}$ and $k_{m,i}$ represent the maximum LMWOM uptake rate and half saturation of LMWOM uptake by bacterial group B_i, respectively. The default values for these parameters are derived from experimental data which observed a growth rate of 7.2 day^{-1} for *Psychromonas psych6C06* on chitin with a $k_{m,i}$ of 7.2 mmol C_{lmwom} m^{-3} [2]. Note, observed bacterial growth rates quantified experimentally using changes in biomass are equivalent to $(\mu_i - m_{lin,i})$ in this model and so $\mu_{max,i}$ is adjusted accordingly (here set to 9.7 day^{-1}). The model is also run with an observed maximum growth rate of 1.2 day^{-1} ($\mu_{max,i}$ = 3.7 day^{-1}) based on a field-based estimate from Liu et al[9] of the growth rate of bacterial species in *Alteromonas* lineage on recalcitrant carboxyl-rich alicyclic molecules (CRAM). $m_{lin,i}$ is set to 2.5 day^{-1} yielding a 25% daily mortality for a growth rate of 9.7 day^{-1}. This is consistent with observed viral mortality rates[10,11]. $m_{lin,i}$ also implicitly accounts for grazing mortality (zooplankton bactivory) and so 25% daily mortality is a conservative estimate of this loss term.

Growth and mortality in the model are limited by temperature ($\gamma_{T,z}$), which is represented as an Arrhenius equation where:

$$\gamma_{T,z} = \tau e^{\left[A_E\left(\frac{1}{T_z + 273.15} - \frac{1}{T_0}\right)\right]} \quad \text{(s9)}$$

T_z is the ambient water temperature at depth z in °C , T_0 is the reference temperature (set as 293.15 K), τ and A_E are unitless coefficients set to 0.8 and -4 x10^3, respectively, after[12]. We use a standard water column temperature profile from[12–14] where:

$$T_z = 12e^{-\frac{z}{150}} + 12e^{-\frac{z}{500}} + 2 \quad \text{(s10)}$$

which results in temperatures decreasing from 18°C at 100 m to 2.2°C at 2,000 m. We test the sensitivity of our results to different temperature limitation formulations (eq. s9) and temperature depth profiles (eq. s10) (Supplementary Material 3).

Bacterial recruitment/departure from the particle and mortality are represented with three terms in the model: a density independent encounter rate (E_i), a detachment rate (L_i), and a density dependent mortality ($m_{lin,i}$). Specifically, $E_{i,z}$ is independent of particle-associated microbial dynamics and rather is a function of the particle size, sinking speed, the motile diffusivity of bacterial cells ($D_{motility}$), and the non-particle associated or free-living community of bacteria in group i (F_i). $E_{i,z}$ (mmol C_{cell} day^{-1} particle^{-1}) is calculated as[15]:

$$E_{i,z} = 4\pi D_{motility} * r_i * Sh_i * F_i * e^{-0.003*(z-100)} \qquad \text{(s11)}$$

Here we use $D_{motility}$ = 5.5 x 10^{-10} m^2 s^{-1} [15]. F_i is an unknown parameter and so is varied over a large range from 10 to 285 cell mm^{-3} [16], which is approximately 1% to 28% of the free-living bacterial community at 100 m assuming an abundance of 1,000 cells mm^{-3}. As the free-living bacterial abundance decreases with depth by approximately an order of magnitude, we similarly scale the encounter rate such that, for a given F_i at 100 m, the encounter rate $E_{i,z}$ decreases exponentially with depth. This results in encounter rates at 100 m which vary between 36 to 1,000 cell minute^{-1} particle^{-1} for a particle with radius of 0.1 cm. By 2,000 m, encounter rates for the same particle size (radius =0.1 cm) are decreased to only 0.12 to 3.4 cells minute^{-1} particle^{-1}).

Laboratory experiments suggest that the detachment rate of bacteria from particles is highly variable and it is generally unknown what dictates this rate of departure in natural environments[2,17,18]. Due to this uncertainty, we represent the net detachment of the bacterial group as a constant rate L_i (day^{-1}). The number of cells which depart the particle is estimated as $L_i Bi$ (mmol C particle^{-1} day^{-1}) and so the absolute number of cells departing the particle increases with larger population sizes – note that the units of B_i are mmol C particle^{-1} not per surface area. Here we set the rate of bacterial net detachment L_i (day^{-1}) to 0.25 day^{-1} based on[2]. We test the sensitivity of our results to this formulation and show that a more complicated density dependent detachment rate produces similar results (Supplementary Material 2 and Supplementary Figure 23).

Finally, we include a density dependent mortality term $m_{lin,i}$. $m_{lin,i}$ was adjusted so as to allow for true extinction on particles with low β_i, as observed in culture[2], here $m_{lin,i}$ = 2.5 day^{-1}. As described above, $\mu_{max,i}$ was adjusted accordingly such that $\mu_i - m_{lin,i}$ was consistent with observed bacterial growth rates.

As each particle sinks and the organic carbon is consumed, the radius of the particle shrinks. Based on in situ observations, we represent particles across a spectrum from dense, carbon rich small particles to larger, 'fluffier', less carbon rich aggregates[19–23] (see Supplementary Material 3 for additional details). Specifically, a large aggregate of radius r_i is assumed to be composed of sub-particles of radius r_s and density $\rho_{particle}$. Here we set $\rho_{particle}$ = 1,230 kg particle m^{-3} [21,23] and estimate that r_s = 14 μm based on a reference aggregate with radius of 1 mm and sinking speed of 12x10^{-4} m s^{-1} [19].

The relationship between the carbon content of particle i (mmol C particle^{-1}) and the radius of particle i (r_i) can then be defined as[19]:

$$C_{poc,i} = \frac{\frac{4}{3}\pi\left(\frac{r_i}{100}\right)^3 \phi f \rho_{particle}}{C_{carbon}} \qquad \text{(s12)}$$

in which, C_{carbon} is molecular weight of carbon (mmol kg^{-1}). ϕ converts from kg particle m^{-3} to kg C m^{-3}. ϕ is estimated using observational data of the organic carbon content of marine particles[20], here set as 0.0494. f represents the fraction of solid material in the aggregate and is estimated as[19,22]:

$$f = A_f \left(\frac{r_i}{r_s}\right)^{D_{fractal}-3} \qquad \text{(s13)}$$

A_f and $D_{fractal}$ are empirical constants that represent the geometry of aggregated particles[19,22]. A_f is unitless and set to 1.49 and $D_{fractal}$ represents fractal dimension, typically ranging from 1 to 3[19]. Here we set $D_{fractal}$ to be 1.85 for particles that are larger than the sub-particle unit ($r_i > r_s$), and 3 for particles that are equal to or smaller than the subunit ($r_i \leq r_s$) (assuming as a solid particle)[19]. This results in a relationship between carbon content and radius which is consistent with empirical estimates (Supplementary Figure 24a).

As the carbon in the particle is consumed by the microbial group, the particle radius is adjusted as (combining equations 12 and 13 and solving for r_i):

$$r_i = \overline{\frac{C_{poc,i}\, C_{carbon}}{\frac{4}{3}\pi A_f r_s^{3-D_{fractal}} \phi \rho_{particle}}}^{\frac{1}{D_{fractal}}} \qquad \text{(s14)}$$

There are multiple ways to define the relationship between particle size and particle sinking speed as suggested in [5,20]. Here we choose to relate size to particle density and calculate sinking using Stokes Law[19]. The sinking speed of each particle (ω_i in m s^{-1}) is thus calculated dynamically as:

$$\omega_i = \frac{g A_f (\rho_{particle} - \rho_{fluid}) \left(\frac{r_i}{100}\right)^{D_{fractal}-1}}{18 \upsilon_{dynamic} \left(\frac{r_s}{100}\right)^{D_{fractal}-3}} \qquad \text{(s15)}$$

where g is the acceleration of gravity of 9.81 m s^{-2}, $\upsilon_{dynamic}$ is the dynamic viscosity of seawater, set to 10^{-3} kg m^{-1} s^{-1}. ρ_{fluid} is the seawater density and set to 1,028 kg m^{-3} [19]. For simplicity, we assume that ρ_{fluid} does not change with depth[23], therefore changes in particle density scale linearly with the specific gravity of the particle. We confirm that our results are not sensitive to our choice for the relationship between size and sinking speed or size and carbon content (Supplementary Material 2, Supplementary Figure 24b).

Model simulations: To understand model dynamics across a wide range of parameter values, we run the model with all combinations of variable particle size at formation (50 μm to 0.4 cm, 7 sizes), particle lability (10 – 1,000 mmol C_{POC} mmol C_{cell}^{-1} day^{-1}, 9 labilities), initial cell density (400-2,800 cell mm^{-2}, 6 initial densities), density of free-living community of bacterial group i (F_i, 10 to 500 cell mm^{-3}, 6 values of F_i), and maximal growth rate of particle-associated bacterial group (1.2 to 7.2 day^{-1}, 2 values of $\mu_{max,i}$). The results from these simulations (n= 4,536) are shown in Supplementary Figure 26.

POC flux analysis: To investigate the impact of particle-associated microbial dynamics on POC vertical flux, we perform a set of stochastic simulations as illustrated in Supplementary Figure 27. Here we simulate a single 2.24 m x 2.24 m water column with a total POC flux at 100 m of 1.5 mol m^{-2} yr^{-1}, consistent with observations[19,24,25]. For each run, the model is initialized at 100 m with the observed particle size distribution using a power law exponent s of -2, -3, or -4[19,26]. Specifically, we resolve 2,256 particles (s= -2), 69,000 particles (s=-3), or 763,615 particles (s=-4). We assume that particle lability is log-normally distributed within each particle size class[27] with a mean lability (β_{avg}) of 100 mmol C_{POC} mmol C_{cell}^{-1} day^{-1} and standard deviation of 0.77 (log scale). All other parameters are assumed to be uniformly distributed. Each particle in the model is then initialized with parameters stochastically chosen from the sets of values described above (Supplementary Table 1). Discretizing the parameters rather than using a continuous range allows us to simulate thousands of particles in a computationally efficient manner while still accounting for stochastic interactions. Specifically, a simulation with continuous parameter values and a particle size distribution of s=-3 (69,000 particles) would require 69 million simulations (69,000 particles * 1,000 stochastic simulations). Rather, our method conducts the same number of stochastic parameter selections but then uses the pre-run simulations with the closest set of parameter values. Extensive tests of the model dynamics indicated that there are no discontinuities in model dynamics across the parameter range suggesting that our 4,536 model runs adequately sampled parameter space (see full description in Supplementary Material 2).

The total POC flux at depth z, $F_{poc,z}$ (mmol C_{POC} m^{-2} day^{-1}), is calculated as the sum of the vertical fluxes of each individual particle as they sink through the water column and are degraded, specifically as:

$$F_{poc,z} = \sum C_{poc\ i,z} N_{i,z} \omega_{i,z} \qquad \text{(s16)}$$

where $N_{i,z}$ is the number of particles of type i per m^3 water column which varies with depth. To simplify the calculation for data analysis, we interpolate the modeled POC flux for each particle type i to a regular 1 m grid.

We run a series of the stochastic experiments with different parameter values: (1) a range of particle size spectra (s=-2, -3 and -4), (2) a range of mean particle lability (β_{avg}=50, 100 and 500 mmol C_{POC} mmol C_{cell}^{-1} day^{-1}), (3) variable growth rates, only high maximum growth rate (μ_{max}= 7.2 day-1) and only low maximum growth rate (μ_{max}=1.2 day^{-1}), (4) variable encounter rates, only low encounter rates (F_i=10-120 cell mm^{-3}) and only high encounter rates (F_i=175-285 cell mm^{-3}). Each set of simulations are run 1,000 times to quantify the full variability in the stochastic simulations. Simulations are run for 600 days which is sufficient for all particles to be fully consumed or exported to >4,000 m. We then calculate the attenuation coefficient b for each run using the least square fit of the power law function and compare our data with the field observation from[24,28].

SUPPLEMENTARY MATERIAL 2: Sensitivity tests

To test the sensitivity of the model results to key assumptions and choices of parameter values, we conducted a suite of sensitivity experiments described below.

Formation depth: We choose 100 m as our particle formation depth as 100 m is classically used as the particle export depth[24,29]. In our default model simulations, the particles are released uniformly at 100 m. However, in reality particle production occurs throughout the upper water column. The depth at which particles are released has important implications for POC degradation rates due to strong vertical gradients in temperature and pressure (which impacts microbial growth and mortality rates), and free-living microbial populations (which impacts particle-cell encounter rate). In order to test the sensitivity of the model to the particle formation depth, we conduct a set of simulations where particles are released at 50 and 75 m. The vertical profiles of temperature and free-living microbial population below 100 m were consistent with the default simulations and then extended to estimate temperature limitation and encounter rate at <100 m depth. The results demonstrate that variable formation depths create substantial variability in the POC flux in upper 500 m, as expected. Capturing this enhanced variability in the upper water column provides an improved match to the observed fluxes from Martin et al[24]. In particular, releasing particles at shallower depths allows faster POC surface consumption (Supplementary Figure 21) due to higher encounter rate and less influence of temperature. However, we see convergence of the model results below 500 m (Supplementary Figure 21). These results suggest that variable formation depth is an important factor in setting the POC flux in the upper ocean (<500 m) and that our model can provide some insight into this process. For example, for a given site, the model can provide a hypothesis as to the depth(s) of formation that best matches the observed POC profiles.

Lability distribution: Here we define lability of POC as an ecosystem property that is a function of the organic matter itself, the bacterial enzymes specific to the bacterial group B_i, and production rate of those enzymes by B_i [27,30]. Previous work has suggested that remineralization rates in natural environments are lognormally distributed [31]. Therefore, we assume that POC lability is log-normally distributed as in[27,30]. However, lability is difficult to quantify in situ, therefore the true nature of this distribution remains unknown. To confirm that the primary conclusions of this study are not driven by the choice of distribution of POC lability, we compare model simulations with a log-normal lability distribution to simulations with a uniform distribution. For consistency, we use the same mean particle lability and range for both simulations ($\beta_i = 20 - 1{,}000$ mmol C_{POC} mmol C_{cell}^{-1} day^{-1}). Both model simulations produce the same shaped POC flux curves demonstrating that our results are not a function of the distribution of particle lability (Supplementary Figure 1). We also test the impact of shifts in the mean lability and show that higher mean lability results in more rapid POC attenuation with depth, as expected (main text Figure 4, Supplementary Figure 28 and Supplementary Figure 29). Finally, we test the impact of the standard deviation of the lability distribution and show that a larger standard deviation yields slightly more variability in the POC profiles but does not significantly change the average of the stochastic distribution.

Single lability particles: To reduce model complexity, free parameters, and computational costs, we assume that each particle consists of a single lability value. In reality, marine particles are complex and made up of many different compounds[32,33]. We also assume that each particle is degraded by a single bacterial group rather than a diverse community. To test the impact of these simplifying assumptions, we run the model with multiple POC lability types per particle (n= 25) and multiple bacterial groups (n= 25), one for each POC type. To allow for a direct comparison to the 'single lability per particle' runs, we apply a log-normal distribution to the POC types co-occurring within a single particle. Specifically, the 'average' lability POC type contributes the largest fraction to the total particle carbon content with log-normally distributed contributions from the high and low-lability fractions. For the sensitivity test, we use a particles radius of 0.2 cm, and maximum growth rate 7.2 day^{-1}. We set the initial bacterial density to 12,800 cell mm^{-2} for all POC types such that the total cells per particle in each bacterial group are also log-normally distributed. To

simplify the simulation, we do not allow for exchange with the free-living bacterial community (bacterial encounter rate = 0 mmol C_{cell} day^{-1} $particle^{-1}$). The multiple-POC simulation is compared against simulation with a single POC type per particle conducted with the same mean lability distributed across 947 particles, same initial radius, bacterial density, maximum growth rate, and encounter rate. Our results show that the single-pool POC (our model) and multiple-pool POC produce similar results (Supplementary Supplementary Figure 2).

Density-dependent bacterial detachment: Micro-fluidic experiments and field measurements estimate a wide range of bacterial departure rates from the particle surface from 0.28 to 43 day^{-1} [2,17,18]. However, it is unknown what dictates this rate. Given the unceratinty in these dynamics, in our model we set the bacterial leave rate to be a relatively low constant rate (0.25 day^{-1}). To test the impact of this assumption, we conduct a suite of sensitivity experiments with a density-dependent formulation of bacterial detachment dynamics where the detachment rate increases as the bacterial density reaches the carrying capacity of the particle. Specifically, in this density-dependent formulation L_i is calculated as a function of the size of bacterial population (B_i, mmol C $particle^{-1}$) where:

$$L_i = \min\left(L_0 + L_{max}\left(1 - \frac{K_i - B_i}{K_i}\right), L_0 + L_{max}\right) \quad \text{(s17)}$$

L_0 is a low background leaving rate (day^{-1}), and L_{max} is the maximum bacterial leaving rate (day^{-1}). These parameters are set as 0.25 and 2 day^{-1}, respectively, based on laboratory measurements[2]. K_i is the carrying capacity of the particle (mmol C_{cell} $particle^{-1}$) and is a function of the particle radius. Here we assume that the maximum cellular density (equivalent to K_i) is mono-surface layer with no penetration such that K_i can be calculated as:

$$K_i = \frac{4\pi r_i^2}{\pi r_{cell}^2} \times \frac{\delta}{12} \quad \text{(s18)}$$

We use a bacterial cell radius (r_{cell}) of 0.62 μm[34,35] and the average bacterial carbon content (δ) of 20×10^{-15} g C $cell^{-1}$ [36]. This density-dependent formulation results in increased detachment rates (L_i) as the bacterial population increases and as the radius decreases. We find that model dynamics are nearly identical for the two different formulations of bacterial detachment rates and produce the same degradation profile of particles with minimal differences (Supplementary Figure 23). This is because once the population is at the carrying capacity, the particle degradation rate is extremely rapid and so the particle is fully consumed very quickly. Sinking speeds are also typically low because the particle is shrinking rapidly and so the impact on the POC flux is negligible. This sensitivity study demonstrates that the model results are not very sensitive to the bacterial detachment formulation and so we use the simplest formulation (constant detachment rate) for the results presented in the main text.

Particle specific gravity and sinking dynamics: There are multiple ways to define the relationship between particle size and its sinking speed, specific gravity, and carbon content. Previous studies have used empirical relationships between particle size and carbon content[20], and particle size and sinking velocity[5]. Alternatively, particle size, specific gravity, and sinking speeds can be related using a more mechanistic formulation (i.e., Stokes law)[19,22]. However, this more mechanistic representation introduces additional model parameters specifically the fractal dimension required to capture large 'fluffy' aggregates. For simplicity, we assume that the reference density does not change following[23] and therefore, changes in specific gravity scale linearly with changes in particle density. To test the sensitivity of our model to our formulation of particle sinking speed and carbon content, we re-run the full range of model simulations (i.e. Fig 3 in the main text) using the particle sinking speeds from[5] and particle density from[20]. Speficically, the sinking speed ω_i (m s^{-1}) for each particle is calculated as:

$$\omega_i = 4.23 r_i^{1.17} \quad \text{(s19)}$$

where r_i is particle radius (m). And the relationship between particle size and carbon content is defined based on [20] as:

$$r_i = \frac{C_{poc,i}}{0.0630704}^{\frac{1}{1.56}} \times 10^{-2} \qquad \text{(s20)}$$

where $C_{poc,i}$ is the carbon content of the particle i in mmol C particle^{-1}. Supplementary Figure 24 compares these different formulations. This sensitivity tests demonstrates that our results were not sensitive to our choice of the relationship between size and specific gravity, or size and carbon content of the particles (Supplementary Figure 24b and Supplementary Figure 25).

***LMWOM Steady-state assumption*:** In our model, low molecular weight organic matter (LMWOM) is produced at the surface of the particle by the enzymatic degradation of polymeric organic matter. LMWOM is then consumed by the particle-associated bacterial population and/or diffuses away from the particle surface. Because both the bacterial LMWOM uptake rate and LMWOM diffusive loss rate are concentration dependent, we track LMWOM at the particle surface in concentration units. Practically, this requires defining the volume around the particle for which this concentration applies. We make the simplifying assumption that, for each time-step in the model, the LMWOM concentration is at steady state such that the production LMWOM is equal to the LMWOM bacterial consumption and diffusive loss (main text eq. 2). We test the validity of this assumption by running a version of the model in which we explicitly resolve the LMWOM gradient around the particle and show that, as long as the volume is small enough, the steady-state calculation produces the same results as the explicit model. The small volume (V in Supplementary Material 1 eq. s2) is defined as:

$$V = \frac{4}{3}\pi[(r_i + d_r)^3 - r_i^3] \qquad \text{(s21)}$$

in which d_r is represent the thickness of the small volume around the particle inhabited by particle-attached bacteria, here is set as 10^{-3} cm. Supplementary Figure 22 shows a comparison between a simulation run using an explicit small volume and small time step and a simulation using the steady-state approximation (main text eq. 2).

SUPPLEMENTARY MATERIAL 3: Model dynamics

Allee effect: The Allee effect refers to the density-dependence of the per capita growth rate in a population where the average growth rate is observed to be lower for small populations compared to larger populations[37]. This effect has been demonstrated in many systems including on experimental particles in the lab and in an individual based model of particle-associated bacteria where growth is impacted by the diffusive loss of nutrients and enzyme away from a chitin hydrogel particles[2] . Here we conduct a series of sensitivity tests to confirm that the Allee effect is also present in our model. When all loss rates, including biological loss from cell detachment and mortality and physical loss from diffusion and advection, are turned off in the model, all populations are able to successfully colonize the particles (Supplementary Figure 6a). As we sequentially add in loss processes (LMWOM diffusive and advective loss and biological loss), we see that a critical threshold develops in which populations below the critical threshold are unable to successfully colonize particles (Supplementary Figure 6b-d). These sensitivity tests further show that particles are consumed faster in the absence of advection, as expected (Supplementary Figure 6c&d). This has important implications for experimental results where consumption rates are estimated in incubation experiments where particles most likely do not experience LMWOM loss due to advection as the particles are not actively sinking during the experiment.

It is also important to note that while the model captures the cooperative degradation that Ebrahimi *et al.* documented[2], the model formulation does not explicitly represent cooperation in the sense that enzyme production is not a function of microbial population size. We show that diffusive loss of LMWOM away from the particle is in of itself a sufficient loss term to impact particle-associated microbial dynamics. We describe an analytical calculation (see Supplementary Material 4) to show that POC degrading bacteria will generically experience linear rather than exponential growth due solely to diffusive loss of LMWOM – in other words, we do not need to invoke cooperation. The calculation also derives the parameter dependence of the "waiting time" before bacterial surface density is built up to a sufficient level to support exponential growth. Typical bacterial parameters generate "waiting time-scales" of several tens of days; see Supplementary Figure 11b. When compounded with mortality and detachment, this density-dependent per capita growth effect gives rise to a hard phase transition allowing colonization only above a threshold density. The value of this threshold is derived in terms of model parameters is plotted in Supplementary Figure 11c

Impact of carbon density and microbial dynamics on degradation rates: As each particle sinks and the organic carbon is consumed, the radius of the particle shrinks. Based on in situ observations, we represent particles across a spectrum from dense, carbon rich small particles to larger, 'fluffier', less carbon rich aggregates[19–23]. As a result, large aggregates degrade faster (more rapid decrease in radius) than small particles (main text Figure 2b F vs. E curve). However, due to their faster sinking speed, large particles reach deeper depths before being completely consumed than small particles (main text Fig 2c, Supplementary Figure 9).

To test the intrinsic relationship between size and degradation rate, we ran a simulation in which we assumed that large particles contained the same carbon density as small particles. Specifically, the carbon content of all particles was assumed to scale linearly with the particle volume as:

$$C_{poc,i} = C_m r_i^{\alpha} \tag{s22}$$

where α is the exponent and here set as 3 (unitless) and C_m is adjusted based on the reference particle of 1 mm radius that has 6.7 μg carbon[5], accordingly set as 0.5583. In this hypothetical case, large particles are denser and so sink faster. These large particles also degrade more slowly than when they are represented as aggregates as there is a higher carbon content (more organic matter per volume). As a result, the large particles end up at significantly deeper depths when represented as solid spheres rather than aggregates, as expected (Supplementary Figure 9). Strikingly, the effect of particle size on degradation rates is secondary to other microbial drivers of degradation rates such as particle lability, encounter rate, and initial bacterial

population (Supplementary Figs. 7 and 8). Specifically, microbial parameters (e.g., particle lability, encounter rate, initial bacterial density) can alter the relative rate of remineralization between particle size classes. For example, while a large particle might degrade faster than a small particle under a certain set of microbial parameters, a shift in lability, encounter rate, or initial bacterial density can result in the small particle degrading more rapidly (Supplementary Figure 8). However, given faster sinking speeds, the larger particle always reaches deeper depths than a smaller particle when both particles have the same parameters.

Size spectra changes over depth: In our model, the attenuation in POC flux with depth is not driven by a shift in particle size spectra with depth (Supplementary Figure 30a). Specifically, our model does not require that small particles are consumed in the surface and large particles persist to depth in order to generate the classic POC flux profile, a result that is consistent with observations[26]. As POC sinks, the labile particles are consumed more rapidly resulting in a shift in the bulk lability towards lower values (Supplementary Figure 31) and a shift towards slower bulk remineralization rates (Supplementary Figure 32). This occurs because bacterial populations consuming higher lability POC are able to more successfully colonize particles and so reach exponential growth more quickly and thus consume labile particles in the surface ocean. Less labile particles are more likely to be associated with struggling communities with lower growth rates and so more likely to persist at depth (Supplementary Figure 30b). It is important to note that while most labile particles are consumed in the upper water column, as expected, some labile particles do persist at depth in the model as a result of the stochastic assignment of microbial dynamics (Supplementary Figure 31 and Supplementary Figure 33). In fact, there is a nearly two orders of magnitude range in remineralization rates supported by any given lability class (Supplementary Figure 30c) (discussed more below).

Impact of discrete vs. continuous parameterization in model dynamics: As described in Supplementary Material 1, in order to reduce computational cost for our stochastic simulations, we sample from a set of discrete values for key model parameters (particle lability, bacterial maximum uptake rate, concentration of free-living community bacteria, and initial bacterial density). This allows us to reduce the number of model simulations needed to represent a particle size spectrum with slope = -3 from 69 million (69,000 individual particles times 1,000 replicates), to 5,000 model simulations. These 5,000 unique profiles are then stochastically resampled to generate a range of water column flux estimates. However, sampling from discrete parameter values rather than a continuous set may result in the model insufficiently sampling parameter space and thus impacting the range of model results. To validate our approach, we compare the output from the model run in both modes using the same range and mean parameter values. The only exception is that we use a slightly wider range of bacterial maximum uptake rates from 1 to 10 day^{-1} that encompasses the two discrete values (1.2 and 7.2 day^{-1}) and slightly higher mean (5.5 day^{-1} vs 4.2 day^{-1}). Due to computational constraints, we conduct this test using only two particle sizes: r=0.02 cm and r=0.4 cm. We find that the two simulations produce the same range of POC flux curves including exponentially growing, struggling, and 'rescue' populations. If we decrease the average particle lability to 65 mmol C_{POC} mmol C_{cell}^{-1} day^{-1} instead of 100 mmol C_{POC} mmol C_{cell}^{-1} day^{-1}, we also capture the same mean and range of POC fluxes (across 1,000 stochastic replicate runs) (Supplementary Figure 3*).* We believe the slight shift in the mean lability for the continuous parameter runs that is necessary to generate the same POC flux as the discrete runs is due to the lognormal distribution used for the lability parameter. Overall, this test demonstrates that discrete parameter values can be used to reduce the computational costs for large-scale stochastic simulations without changing the model dynamics.

Impact of Temperature: Previous work has suggested that temperature may play an important role in determining the efficiency of POC transfer[26,38,39]. However, there is no clear observational evidence of a systematic relationship between the POC flux and temperature, and often contrasting results have been reported[40]. In our model, growth and mortality are both limited by temperature, following previous studies[13,30,41]. As such, temperature plays a role in the microbial 'loss' processes. Specifically, because temperature acts to reduce growth rates it becomes more challenging for communities to overcome the other

loss terms. To isolate the impact of temperature on the model results, we conduct a set of model simulations in which the temperature impact is varied. The default temperature limitation formulation (eq. s9) has been used previously[14]. We compare this with an enhanced temperature limitation formulation from [23]. These two temperature limitation formulations span the observed temperature dependent decrease in growth rate for marine heterotrophic bacteria from both published[23,42–44] and unpublished (Supplementary Table 2) studies (Supplementary Figure 5). In addition to assessing the impact of enhanced temperature limitation, we also analyze the impact of removing temperature limitation ('Temperature off' simulations), and of different temperature profiles.

We compare the results of the 'Temperature off' simulations to the default model results ('Temperature on'). Without temperature limitation, we see faster consumption rates for particles that are fully consumed in the upper water column, as expected. However, bacterial populations that struggle to colonize particles exist in the model even when temperature limitation is removed. These simulations suggest that struggling populations are more sensitive to temperature limitation than populations above the critical threshold which can attain high growth rates despite temperature limitation (Supplementary Figs. 4 & 30b). In addition, particles that are large at formation are more impacted by temperature limitation as they reach greater depths where temperature limitation is greater (Supplementary Figure 7). However, they are also more likely to be associated with populations at depth that have surpassed the critical threshold and so growing exponentially (Supplementary Figure 30). Overall, the model dynamics remain fairly unchanged (Supplementary Figure 18).

Next, we run the model using a larger temperature-associated decrease in growth rate such that growth rate decreased by 80% in the upper 2,000 m consistent with the formulation from[23]:

$$\gamma_{T,z} = \tau e^{\left[2A_E\left(\frac{1}{T_z+273.15}-\frac{1}{T_0}\right)\right]} \quad \text{(s23)}$$

Equation s23 is used instead of eq. s9, which produces a decrease of 55% by 2,000 m. At 200 m, the differences in the modeled growth rates between the two simulations with different temperature limitation formulations could be explained simply by the differences in temperature limitation (Supplementary Figure 34). By 500 m, we see that more populations have reached exponential growth in the milder-temperature limitation simulations (overcome diffusive loss of LMWOM) than in the enhanced-temperature limitation simulations (reduced growth due to lower LMWOM concentrations) (Supplementary Figure 34). However, despite these differences in growth rates, we see that water column POC fluxes are very similar between the two runs (Supplementary Figure 20). We attribute this to the fact that the model dynamics are not fundamentally changed: populations that are able to successfully colonize particles and grow exponentially are able to still do so and the change in maximum growth rate due to temperature limitation does not significantly alter the depth at which the particle is completely consumed. When populations are struggling, their growth rates are low in both simulations, the consumption of the particle is therefore also slow, and the particle persists.

Finally, to simulate a different oceanographic region, we run the model using a narrower temperature gradient from 12°C to 3°C (0-2,000 m) based on a temperature profile from the subpolar North Atlantic region (CCHDO Hydrographic Cruise 64PE20000926). We find the same degree of variability of POC flux captured in both simulations (Supplementary Material 3, Supplementary Figure 19).

Variable remineralization rates: The conversion of particulate organic matter into CO_2 (remineralization) is typically represented as a single rate processes in biogeochemical models rather than explicitly represented as a dynamic microbial process[19,39,45]. In this study, particle remineralization rates are an emergent property of the model and vary as a function of particle-associated microbial dynamics. As a result, in our simulations, particle remineralization rates vary by several orders of magnitude across particles and over depth (Supplementary Figs. S12 and S13). This variability in remineralization rates is related to whether a bacterial population is struggling to successfully colonize a particle (low

remineralization rates) or is successfully colonizing a particle (high remineralization rates). Thus, variable remineralization rates are intrinsically linked to the model dynamics described in the main text. Furthermore, variable remineralization rates generate variable particle residence times within the water column. The residence time of particles in the model ranges from a few days (~5 days) to several months (~200 days) depending on the biotic and abiotic parameters with most particles lasting for a few weeks (~30 days, lognormally distributed) below the export depth, consistent with observations[46,47]. We believe that this provides strong evidence for the need to include such dynamics in global biogeochemical models. Our model also provides predictions of how particle associated microbial dynamics might vary with depth and location which can be tested experimentally.

Direct uptake of POC: The default model formulation focuses on POC degradation through extracellular enzymatic cleavage of polymers into smaller compounds (LMWOM) as we believe this is a dominant mode of organic matter degradation[48–53]. However, the default model does not capture either the dynamics of degraders who are able to directly take up polymers and degrade them intercellularly (i.e., selfish-uptake[54–56]) or of degraders caught within the matrix of a particle where diffusive loss would be minimized. To understand the impact that this alternative uptake strategy might have on particle degradation and the findings of our paper, we have developed a modified version of the model that simulates direct uptake by the degraders (i.e., no LMWOM is generated). We modify equations s1 and s8 to:

$$\frac{dC_{poc,i}}{dt} = -\xi_i B_i \quad \text{(s24)}$$

$$\mu_i = \mu_{max,i}\gamma_{T,z} \quad \text{(s25)}$$

where ξ is the carbon uptake rate by the microbial degrader (mmol C_{POC} mmol C_{cell}^{-1} day^{-1}). ξ is constrained by the rate at which microbes can incorporate carbon into their biomass (i.e., microbes cannot degrade the particle faster than they can grow) and so is set to $\xi = \mu_i/y$, where y is the growth yield (here set to 0.2). For these runs, we varied $\mu_{max,i}$ from 0.2 to 10 day^{-1}. We run this model using a similar range of parameter values (initial biomass, free living concentration, particle size, etc.) and same temperature limitation as the default model simulations.

We demonstrate that the three types of particle degradation profiles also emerge for direct-uptake degraders: 1) exponential growth and rapid consumption, 2) populations struggling to overcome loss (here only mortality and detachment rate), and 3) rescued populations due to encounter with free-living bacterial communities (as shown in Supplementary Figure 15). Of note, the 'rescued' populations are more common in the enzymatic cleavage model than in the direct uptake model.

These additional simulations confirm the importance of populations struggling to overcome loss processes. In the case of direct-uptake, the losses are simply mortality and detachment. The primary finding of our work remains unchanged: POC consumption is temporally and spatially variable as a function of microbial growth dynamics and microbial community composition. Changes in microbial processes (e.g., enzyme kinetics, loss rates) have the potential to significantly alter the rate of particle degradation and thus the POC flux. This is true for both extracellular cleavage of polymers and for direct-uptake.

SUPPLEMENTARY MATERIAL 4: Density-dependent growth on the surface of particulate organic carbon (POC)

As described in Supplementary Material 3, the model described in the main text captured the cooperative degradation of POC that Ebrahimi *et al*. documented[2]. Specifically, we found through numerical simulation that diffusive loss of LMWOM away from the particle is in of itself a sufficient loss term to impact particle-associated microbial dynamics (Supplementary Figure 6). Here we present an analytical calculation to show that degradation of POC by a layer of cells at the surface leads to sub-exponential (2nd order) rather than exponential growth due solely to the diffusive loss of LMWOM. When combined with detachment and mortality, these dynamics lead to a phase transition where growth on POC occurs only if the number of cells on a particle exceeds a threshold. Importantly, this calculation highlights that no direct cooperative interaction is necessary for the observed result of strongly cooperative POC degradation and bacterial growth. The analytical results derived here also provide insight into how the very long (multiple week) time scale of POC degradation can arise, and how the critical cellular density (cells per surface area) needed for successful colonization of POC depends on microbial and physical parameters (e.g., POC degradation rate, mortality, and maximum growth rate).

We consider the synthesis and consumption of nutrient (e.g., N-acetyl glucosamine, or GlcNAc) of concentration $C_{lmwom}(\vec{r},t)$ by bacteria adhering to the surface of POC centered at $\vec{r}=0$. We can describe the nutrient dynamics by the diffusion equation:

$$\frac{\partial}{\partial t} C_{lmwom} = D\nabla^2 C_{lmwom}(\vec{r},t) + s(\vec{r},t) \qquad \text{(s26)}$$

where D is the nutrient diffusion coefficient and $s(\vec{r},t)$ is the net nutrient secretion rate, which is the difference between the rate of nutrient synthesis and uptake. Here, for simplicity, we neglect the effect of fluid flow. The impact of advective loss due to flow around a falling particle can be included by changing D to $Sh \cdot D$ where Sh is the Sherwood number as in eq. s3.

We consider a uniform layer of $B(t)$ bacterial cells spread on the surface of a spherical POC of radius R. This can be described as a layer of bacteria of density $\rho(\vec{r},t) = \sigma(t)\delta(r-R)$, where δ is the Dirac delta function, $r \equiv |\vec{r}|$ is the radial distance from the center of POC, and $\sigma(t) \equiv B(t)/(4\pi R^2)$ is the surface cell density in units of cells per surface area. The nutrient secretion rate $s(\vec{r},t)$ in main text eq. 1 can then be expressed as

$$s(\vec{r},t) = [\beta - \mu(C_{lmwom}(\vec{r},t))/y_{lmwom}] \cdot \rho(\vec{r},t) \qquad \text{(s27)}$$

where β is the specific nutrient synthesis rate (as defined in the *main text* eq. 1), y_{lmwom} is the biomass yield of the nutrient, and

$$\mu(C_{lmwom}) = \mu_{max} \frac{C_{lmwom}}{C_{lmwom} + k_m} \qquad \text{(s28)}$$

is the Monod formula for the bacterial growth rate (as in eq. s8), with μ_{max} being the saturated growth rate and k_m being the Monod constant.

Assuming that nutrient dynamics reaches a stationary state at much faster time scale than the changes in cell density and further taking spherical symmetry, we can set $\partial C_{lmwom}/\partial t$ in eq. s26 to zero, yielding

$$\frac{1}{r^2}\frac{d}{dr}\left(r^2\frac{d}{dr}C_{lmwom}\right) = -\frac{\mu_{max}\sigma}{y_{lmwom}D}\delta(r-R)\left[\tilde{\beta} - \frac{C_{lmwom}(r)}{C_{lmwom}(r) + k_m}\right] \qquad \text{(s29)}$$

where $\tilde{\beta} \equiv \beta y_{lmwom}/\mu_{max}$ is a dimensionless parameter describing the ratio of the nutrient synthesis rate and the maximum uptake rate. Performing one integral over r (to get rid of the δ-function), we obtain:

$$r^2\frac{d}{dr}C_{lmwom} = \begin{cases} 0 & \text{if } \ 0 < r < R \\ -\frac{\mu_{max}\sigma R^2}{y_{lmwom}D}\left[\tilde{\beta} - \frac{C_{lmwom}(R)}{C_{lmwom}(R) + k_m}\right] & \text{if } \ r > R \end{cases} \qquad \text{(s30)}$$

One more integral leads to

$$C_{lmwom}(r) = \begin{cases} C_{lmwom}(R) & \text{if } \ 0 < r < R \\ \frac{\mu_{max}\sigma R^2}{y_{lmwom}Dr}\left[\tilde{\beta} - \frac{C_{lmwom}(R)}{C_{lmwom}(R) + k_m}\right] & \text{if } \ r > R \end{cases} \qquad \text{(s31)}$$

such that

$$C_{lmwom}(R) = \sigma\frac{\mu_{max}R}{y_{lmwom}D}\left[\tilde{\beta} - \frac{C_{lmwom}(R)}{C_{lmwom}(R) + k_m}\right] \qquad \text{(s32)}$$

eq. s32 is exactly eq. 2 of the main text, with the surface cell density being $\sigma = B/(4\pi R^2)$ where B is the number of bacteria on the particle, and with the diffusive loss rate being $d_{loss} = 4\pi R \cdot D$ (eq. s3) for $Sh = 1$. Denoting the concentration of LMWOM at the particle surface ($C_{lmwom}(R)$) by C_0 for simplicity, we can rewrite the above equation as

$$\frac{C_0}{k_m} = \frac{B}{B_m}\left[\tilde{\beta} - \frac{C_0}{C_0 + k_m}\right] \qquad \text{(s33)}$$

where $B_m \equiv 4\pi R \cdot D y_{lmwom} k_m/\mu_{max}$.

Eq. s33 is the central equation relating the substrate concentration and bacterial density. Solution of this equation yields the relation $C_0(B)$ – the concentration of LMWOM at the particle surface as a function of surface cell density – which when inserted into the Monod growth law (main text eq. 2), gives the density-dependent growth rate, $\mu_0(B) \equiv \mu(C_0(B))$. Eq. s33 contains two parameters, $\tilde{\beta}$ and B_m. Since $\frac{C_0}{C_0+k_m} \leq 1$, there are two regimes depending on whether $\tilde{\beta}$ is larger or smaller than 1.

For $\tilde{\beta} \gg 1$, uptake is negligible, and we have

$$\frac{C_0}{k_m} \approx \tilde{\beta}\frac{B}{B_m} \qquad \text{(s34)}$$

This leads to the result

$$\mu_0(B) \approx \mu_{max}\frac{B}{B + B_m/\tilde{\beta}} \quad \text{for } \ \tilde{\beta} \gg 1 \qquad \text{(s35)}$$

with an effective 'Michaelis constant' for density-dependent growth, $B_m/\tilde{\beta} = 4\pi R k_m D/\beta$.

In the limit $\tilde{\beta} < 1$, i.e., nutrient synthesis rate is small. Since the right-hand side of eq. s33 must be positive, we must have

$$\frac{C_0}{k_m} < \frac{\tilde{\beta}}{1-\tilde{\beta}} \tag{s36}$$

This is a simple statement that if the nutrient synthesis rate is smaller than maximum uptake rate, then the nutrient concentration on the surface must necessarily decrease to match the two. For small β (corresponding to substrate that are difficult to breakdown e.g., for chitin degradation), we expect to have $\tilde{\beta} \ll 1$, and consequently $C_0 \ll k_m$. This imposes a limit on the growth rate:

$$\mu_0 \approx \mu_{max}\frac{C_0}{k_m} < \mu_{max}\frac{\tilde{\beta}}{1-\tilde{\beta}} \approx \beta y_{lmwom} \tag{s37}$$

i.e., cells cannot grow at a faster rate than the substrate is synthesized.

The expressions (s36) and (s37) do not yet take into account of diffusion, which should make growth even slower. To include diffusion, we solve eq. s33 approximating the Michaelis term by C_0/k_m (since $C_0 \ll k_m$ for $\tilde{\beta} \ll 1$). Then we obtain

$$\frac{C_0}{k_m} \approx \frac{\tilde{\beta}\,B}{B+B_m} \tag{s38}$$

and the bacterial growth rate becomes

$$\mu_0(B) = \mu_{max}\frac{\tilde{\beta}\,B}{B+B_m} \quad \text{for} \quad \tilde{\beta} \ll 1 \tag{s39}$$

with $\mu_{max}\tilde{\beta} = \beta \cdot y_{lmwom}$.

Eqs. s35 and s39 describe the density-dependent growth rate $\mu_0(B)$ for two extreme ranges of the parameter $\tilde{\beta} \equiv \beta y_{lmwom}/\mu_{max}$.

It is interesting to note that at low density, both expressions give the limiting expression

$$\mu_0(B) \approx \mu_{max}\tilde{\beta}B/B_m \quad \text{for} \quad B \to 0 \tag{s40}$$

For small $\tilde{\beta}$, the growth rate saturates to $\mu_0(B) \approx \mu_{max}\tilde{\beta}$ as σ increases beyond B_m, whereas for large $\tilde{\beta}$, the growth rate saturates to $\mu_0(B) \approx \mu_{max}$ as B increases beyond $B_m/\tilde{\beta}$. The two forms for small and large $\tilde{\beta}$ (i.e., eqs. s35 and s39) can be captured by the following approximate expression,

$$\mu_0(B) \approx \frac{\mu_{max}\tilde{\beta}}{\tilde{\beta}+1}\frac{B}{B+B_m/(\tilde{\beta}+1)} \tag{s41}$$

These behaviors are seen in Supplementary Figure 32a from numerical solution of eqs. s28 and s33.

We next turn to the population dynamics resulting from the density-dependent growth rate $\mu_0(B)$, given by the equation

$$\frac{dB}{dt} = \mu_0(B) \cdot B \qquad \text{(s42)}$$

with the initial condition $B_{init} \equiv B(0)$. For small B where $\mu_0(B)$ is given by eq. s40, the dynamics is 2nd-order, with the solution

$$B(t) = \frac{B_{init}}{1 - \tilde{\beta}\mu_{max}(B_{init}/\sigma_m)t} \approx B_{init} \cdot \left(1 + \tilde{\beta}\mu_{max}\left(\frac{B_{init}}{B_m}\right)t\right). \qquad \text{(s43)}$$

Thus, the density increases approximately linearly in time until a time

$$t_w = \frac{B_m}{B_{init}\,\tilde{\beta}\mu_{max}} \qquad \text{(s44)}$$

when $B(t)$ starts to increase nonlinearly to quickly reach the density where μ_0 saturates and exponential growth takes over. Note that eq. s44 for t_w is valid as long as the linear approximation of $\mu_0(B)$ is valid. For $\tilde{\beta} \gg 1$, this is true for $B_{init} \ll B_m/\tilde{\beta}$ (Eq. (24)), or $t_w \gg \mu_{max}^{-1}$. For $\tilde{\beta} \ll 1$, this is true for $B_{init} \ll B_m$ (eq. s38), or $t_w \gg \left(\tilde{\beta}\mu_{max}\right)^{-1}$. Results from direct numerical calculation of the waiting time is shown in Supplementary Figure 32b. We see that for μ_{max} of the order of 1/h, the waiting time can be several thousand hours if $\tilde{\beta}$ and/or B_{init}/B_m is small.

Since the smallest number of cells possible to be on a particle is 1, we can estimate the lower bound of B_{init}/B_m as when $B_{init} = 1$. For the chitin degrading *Vibrio* sp. 1A01 with nutrient being GlcNAc monomers, $y_{lmwom} \approx 10^9\, cells/ml/5mM$ GlcNAc and $k_m \approx 1\,\mu M$, such that $y_{lmwom}k_m \approx 2 \times 10^5\, cells/ml$. For particles of radius $R = 100\,\mu m$ and using typical diffusion coefficient for sugars, $D = 500\,\mu m^2/s$, we find $B_m \approx 500\, cells$, so that $B_m/B_{init} < 500$. Further, recent measurements indicate that $\tilde{\beta} \lesssim 1$. Then eq. s44 yields $t_w \approx 1000\, h$ or 40 days. For arbitrary initial inoculant density and POC radius, we have

$$t_w \approx \frac{40\, days}{B_{init}} \cdot \frac{R}{100\mu m} \qquad \text{(s45)}$$

Of course, if the initial cell number on particle is truly as low as $B_{init} = 1$, then the continuum approximation used in formulating the model, particularly the assumed uniform layer of cells with surface density σ, would not be valid. A reasonable restriction for the applicability of this model may be $B_{init} \geq 10$. In general, B_{init} will be dependent on the particle size R, and consequently, t_w could increase or decrease with the particle size depending on the detailed dependence of B_{init} on R. In the simplest case where B_{init} is proportional to the cell-particle encounter rate, which is itself proportional to the radius of POC (see eq. s11), then t_w is expected to be independent of the particle size – recapitulating the results from the dynamic model described in the main text which show that size alone does not determine remineralization timescales.

In summary, this calculation shows that 1) a density-dependent growth effect occurs naturally and generically, without assuming any cooperative interaction, due to the diffusive loss of nutrients away from the particle, and 2) the time scale to overcome this density-dependent effect is of the order of several tens

of days if a particle starts with just a few cells. When this density-dependent effect on growth is further compounded with mortality (of rate m_{lin}) and detachment or loss (of rate L), e.g., eq. s7,

$$\frac{dB}{dt} = (\mu_0(B) - m_{lin} - L) \cdot B \quad \text{(s46)}$$

then a strong Allee effect (i.e., a phase transition) occurs where a population on the particle would only survive and grow if the initial population size is above a critical threshold B_c. The fact that the waiting time is easily the order of several days (eq. s45) and the typical death rate of marine bacteria is of the order of a few days suggests that this strong Allee effect is truly relevant for realistic parameters. If we insert the expression in eq. s41 for $\mu_0(B)$ in eq. s46, then the critical threshold, defined for $\frac{dB}{dt} = 0$ or $\mu(B_c) = m_{lin} + L$, becomes

$$B_c = B_m \frac{m_{lin} + L}{\tilde{\beta}\, \mu_{max} - (m_{lin} + L)(\tilde{\beta} + 1)} \quad \text{(s47)}$$

With $m_{lin} + L \approx 0.1/h$ (Supplementary Table 1), we have $B_c \approx 0.12\, B_m \approx 60\ cells$ for $\tilde{\beta} = 1$ and $B_c \approx 0.75 B_m \approx 400\ cells$ for $\tilde{\beta} = 0.3$ using the above-mentioned parameters for *Vibrio* sp. 1A01.

Supplementary Table 1. List of model parameter values

Symbol	Parameter	Value(s)	Reference
r_0	Particle radius	50, 100, 200, 500, 1000, 2000, 4000 μm	[26]
β	Particle lability	10, 18, 32, 56, 100, 180, 315, 560, 1000 mmol C_{POC} mmol C_{cell}^{-1} day^{-1}	Assume
y_{lmwom}	Aerobic bacterial growth efficiency on low molecular weight organic matter (LMWOM)	0.2 mmol mmol C_{cell} C_{lmwom}^{-1}	[2]
D	Molecular diffusion coefficient for the chemical solute (glucose) in water	6 x 10^{-6} cm^2 s^{-1}	[3]
$\upsilon_{kinematic}$	Kinematic viscosity of seawater	10^{-2} cm^2 s^{-1}	[7,8]
m_{lin}	Bacterial mortality rate	2.5 day^{-1}	
μ_{max}	Maximum growth rate	3.7 and 9.7 day^{-1}	[2,9]
k_m	Half saturation of bacterial LMWOM uptake	7.2 mmol C_{lmwom} m^{-3}	[2]
A_E	Temperature modification regulator	-4 x10^3, unitless	[12]
T_0	Reference temperature	293.15 K	[12]
τ	Temperature coefficient	0.8, unitless	[12]
$D_{motility}$	Motile diffusivity of bacterial cells	5.5 x 10^{-10} m^2 s^{-1}	[15]
F	Concentration of non-particle associated or free-living community of bacteria	10, 65, 120, 175, 230, 285 cell mm^{-3}	[16]
L_0	Low background leaving rate	0.25 day^{-1}	[2]
r_{cell}	Bacterial cell radius	0.62 μm	[34,35]
σ_{init}	Initial bacterial cell density<br>$\sigma_{init} = \frac{B_{init}}{4\pi r_0^2}$	400, 880, 1360, 1840, 2320, and 2800 cell mm^{-2}	[2]
s	Particle size spectra power law exponent	-4, -3, -2, unitless<br>$n_{particle} = d^n$ xΔd, in which $n_{particle}$ is the number of particles per volume, d is particle diameter, Δd is the diameter range	[19,26,57]
δ	Average bacterial carbon content	20 x 10^{-15} g C cell^{-1}	[36]
g	Acceleration of gravity	9.81 m s^{-2}	[19]

A_f	Empirical constant that represents the geometry of aggregated particles	1.49 unitless	[19,22]
$\rho_{particle}$	Universal particle density	1,230 kg m^{-3}	[21,23]
ρ_{fluid}	Seawater density	1,028 kg m^{-3}	[19]
$D_{fractal}$	Fractal dimension	1-3 (unitless)<br><br>Here set as 1.85 for $r_i > r_s$, and as 3 for $r_i < r_s$	[19]
$\upsilon_{dynamic}$	Dynamic viscosity of seawater	10^{-3} kg m^{-1} s^{-1}	[19]
r_s	Radius of sub-particle	14 μm	[19]
ϕ	Constant coefficient adjusted using the observational data for carbon content of the average marine particles with[20] to account for non-organic carbon fraction of the particle	0.0494	[19,20]
C_{carbon}	Molecular weight of carbon	12 x 10^6 mmol kg^{-1}	

Supplementary Table 2. Growth rates of *Vibrio* sp. 1A01 in different temperatures. *Vibrio* sp. 1A01 was isolated from chitin enrichments as described in[58]. Cultures were grown in glucose minimal medium as described in[59] ('strongly buffered HEPES minimal growth medium') with two modifications: trace metal concentrations were 5x less and vitamins were included. Growth rates were measured following the protocol described in[59].

**Temperature (°C)**	**Growth rate (hr^{-1})**	
	Marine Broth	Glucose minimal medium
15	0.54	0.30
20	0.83	0.54
23.5	1.03	0.59
25	1.17	0.67
27	1.31	0.69
27.5	1.14	0.64

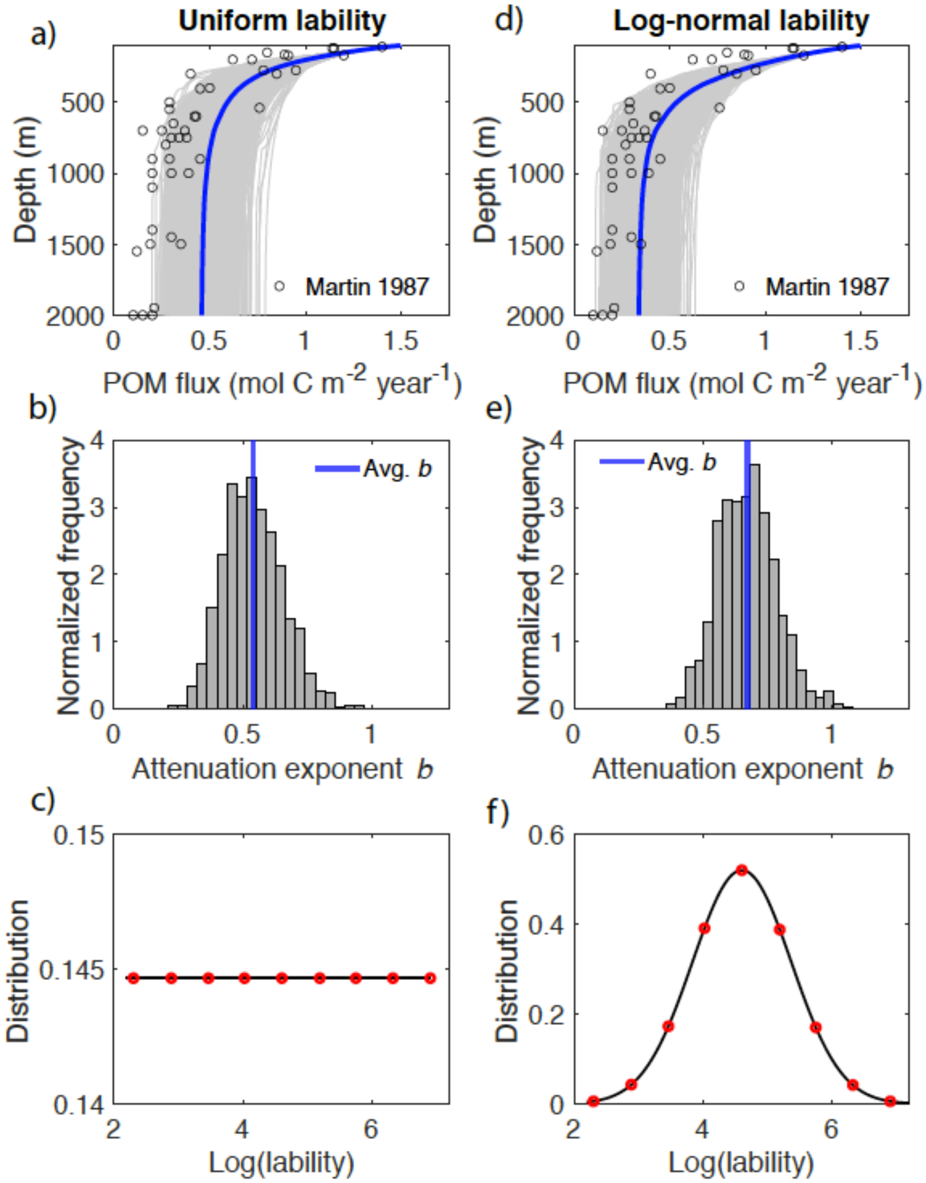

Supplementary Figure 1. Particulate Organic Matter (POM) vertical dynamics resulting from a uniform particle lability distribution (a, b, c) and a log-normal distribution with a mean lability of 100 mmol C_{lmwom} mmol C_{cell}^{-1} day^{-1} (d, e, f). Total POM flux over depth for 1,000 stochastic simulations is shown **(a, d)**. Each gray curve is a single model experiment and is the sum of 69,000 randomly initialized particles. The blue curve represents the average of the 1,000 gray curves. Open circles represent observed POM fluxes from the North Pacific[24]. Normalized distribution of attenuation coefficients for 1,000 stochastic simulations is plotted **(b, e)**. The blue line is the mean attenuation value for our 1,000 simulations. Particle lability distribution for each simulation is also given **(c, f)**.

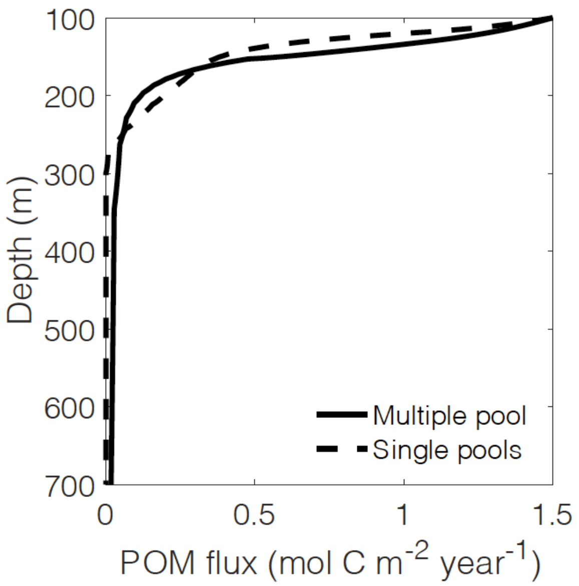

Supplementary Figure 2. Model dynamics with multiple particulate organic matter (POM) pools. Comparison between model simulations run with multiple-POM pools in a single particle (solid line) and multiple particles each with a single POM pool (dashed line) This sensitivity test (described in Supplementary Material 2) uses a particle radius of 0.2 cm, maximum growth rate of 7.2 day^{-1}, initial bacterial density of 12,800 cell mm^{-2} for all POM types, and assumes no exchange with the free-living bacterial community (bacterial encounter rate = 0 mmol C_{cell} day^{-1} $particle^{-1}$).

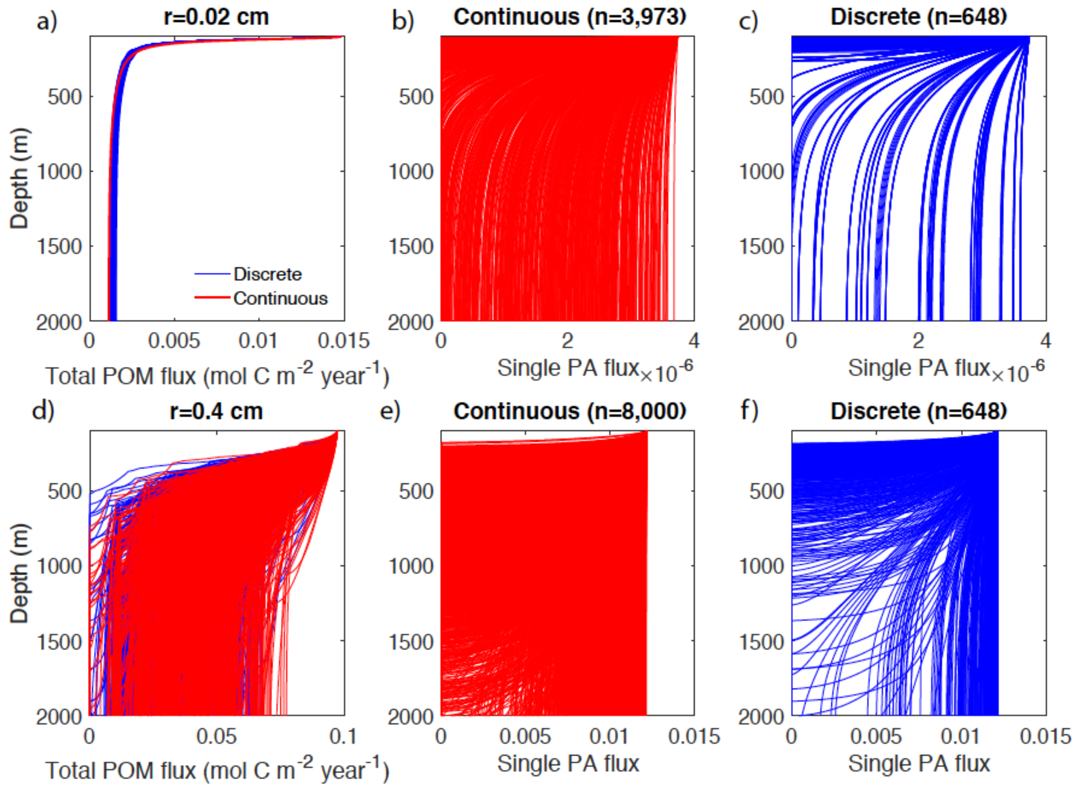

Supplementary Figure 3. Discrete vs continuous parameterizations: Model output is shown for small particles r=0.02 cm (a, b, c) and large particles r= 0.4 cm (d, e, f) with particle size spectra power law exponent *s*=-3. The total particulate organic matter (POM) flux is the sum of 3,973 small particles (a) or 8 large particles (d). For the continuous parameterization simulations (red curves), parameter values are stochastically sampled over the given ranges for each particle (N=3,973 for small particles and N=8 for large particles). For the large particles, this is iterated 1000 times (total model simulations = 8,000) and the results are shown in panels d and e. Given the computational costs, only a single set was conducted for the small particles (total model simulations N =3,973) (a, b). The simulations with discreate parameterization (blue curves) were conducted as described in the main text. Specifically, for each particle size, 648 individual particle simulations were conducted consisting of every possible discrete parameter combination (panels c and f). These results were then stochastically resampled to generate a set of 3,973 individual small particles or 8 large particles and the total POM flux was calculated as the sum of these individual dynamics. This resampling was repeated 1000 times for both the small and large particles. These runs are described in Supplementary Material 3.

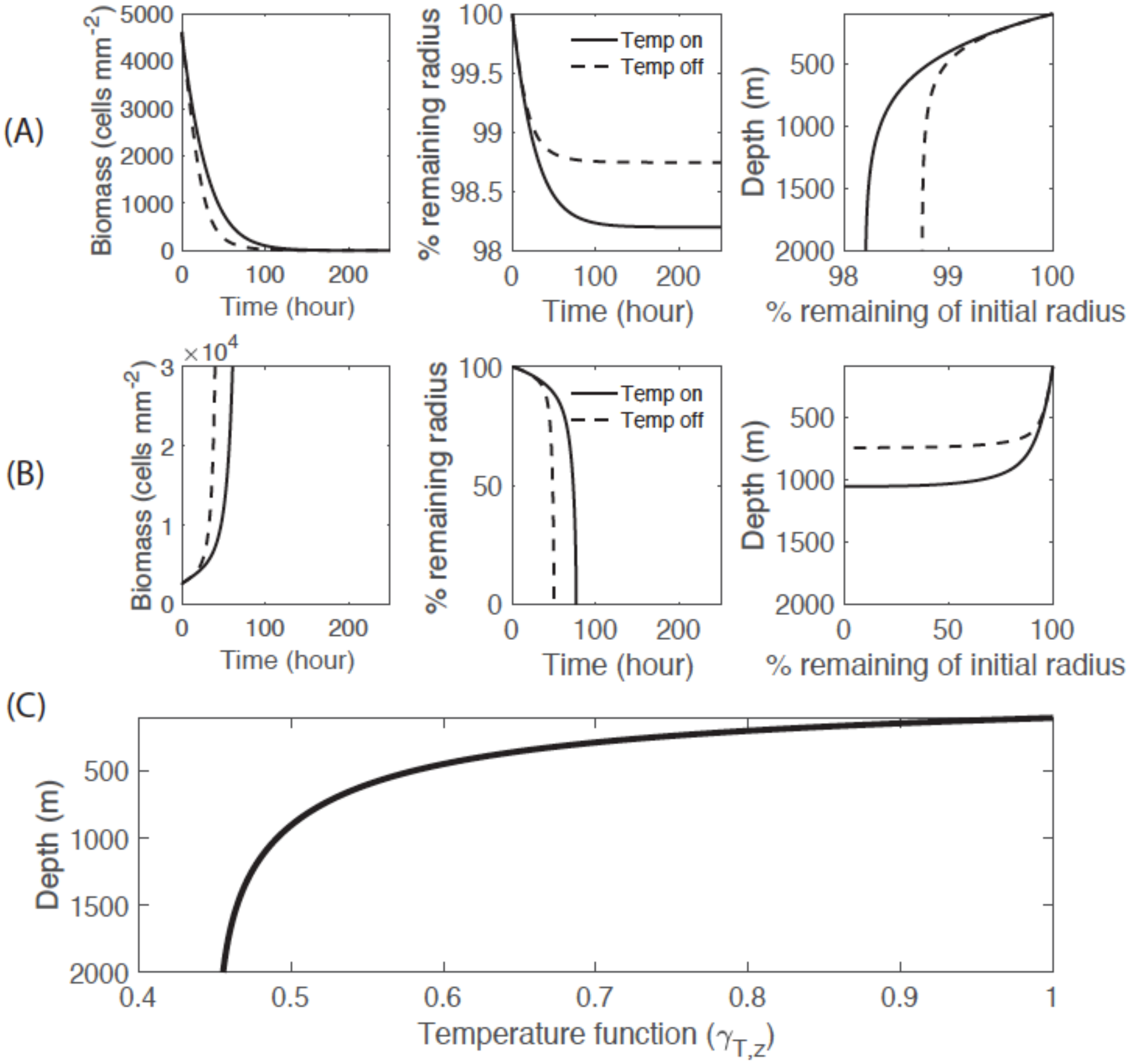

Supplementary Figure 4. Temperature dependence of vertical dynamics. Model dynamics for a single particle type with a struggling microbial population **(A)** and thriving microbial population **(B)**. Simulations with and without the impact of temperature are shown **(A, B).** Panel **(C)** plots the depth dependent temperature function ($\gamma_{T,z}$, Supplementary Material 1, eq. s9).

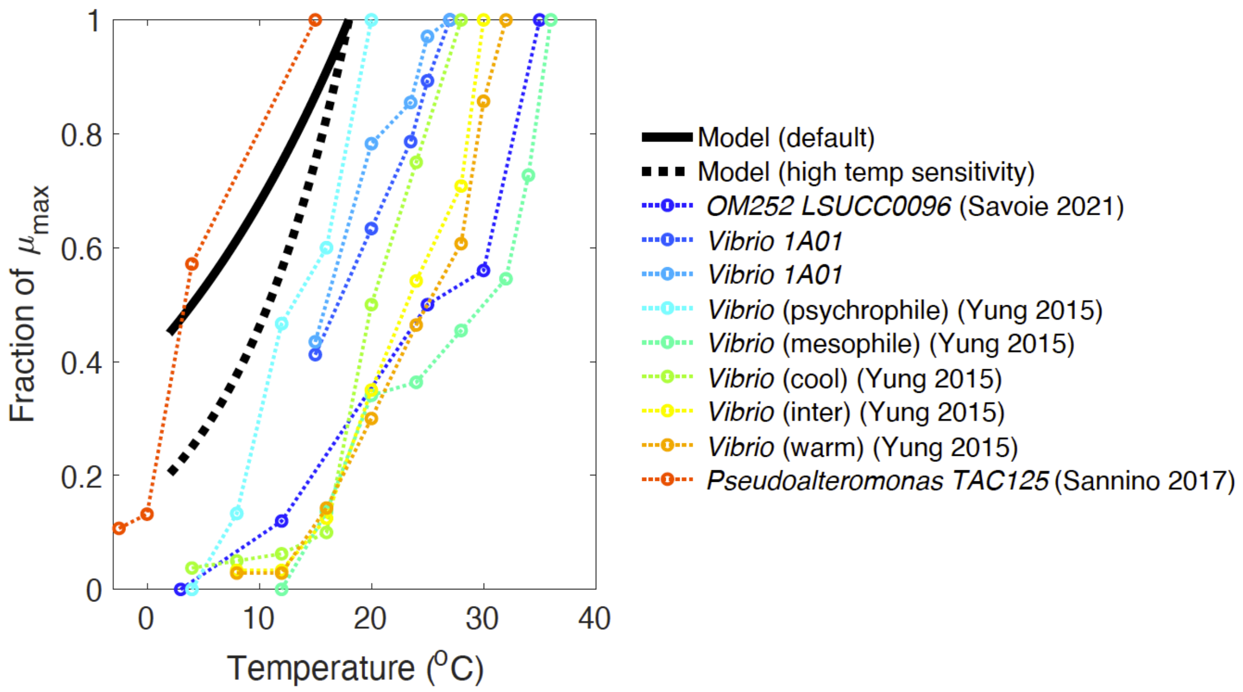

Supplementary Figure 5. Thermal performance curves of cultured heterotrophic marine bacteria as compared to the modeled temperature limitation functions. The y-axis represents the fraction of maximum growth rate (μ_{max}) of different bacteria isolates over a range of temperatures (in Celsius).

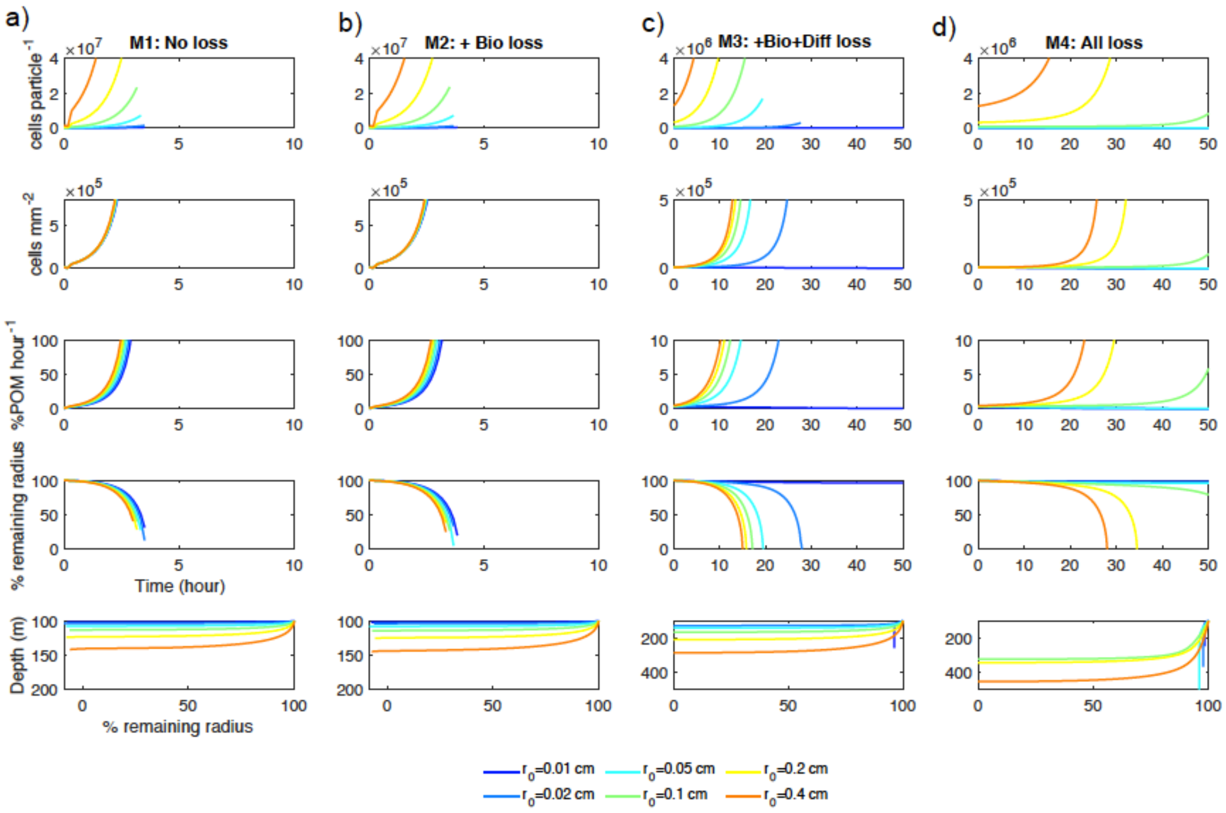

Supplementary Figure 6. Allee Effect in the model. To test the influence of loss processes on particle organic matter (POM) degradation rates, 4 sets of model simulations were conducted: (a) M1: All biological (cell mortality and detachment) and physical (diffusion and advection) loss processes are turned off ($m_{lin,i} = 0, L_i = 0, d_{loss,i} = 0$), (b) M2: Biological loss rates are turned on while physical loss rates are turned off ($m_{lin,i} = 2.5\ day^{-1}, L_i = 0.25, day^{-1},\ d_{loss,i} = 0$), (c) M3: Biological loss rates and diffusive loss are turned on while advective loss is turned off ($m_{lin,i} = 2.5\ day^{-1}, L_i = 0.25, day^{-1}, Sh_i = 1$), (d) M4: Biological loss rates and physical loss rates are turned on ($m_{lin,i} = 2.5\ day^{-1}, L_i = 0.25, day^{-1}, Sh_i = f(r_i)$ as describe in eq. s4). All model simulations are done with a range of particle sizes (r_0=0.01 to 0.4 cm) with no exchange between particles and free-living bacteria and no temperature impact on bacterial growth rate. A particle lability of 150 mmol C_{POM} mmol C_{cell}^{-1} day^{-1} and initial bacterial population of 3,200 cells mm^{-2} are used.

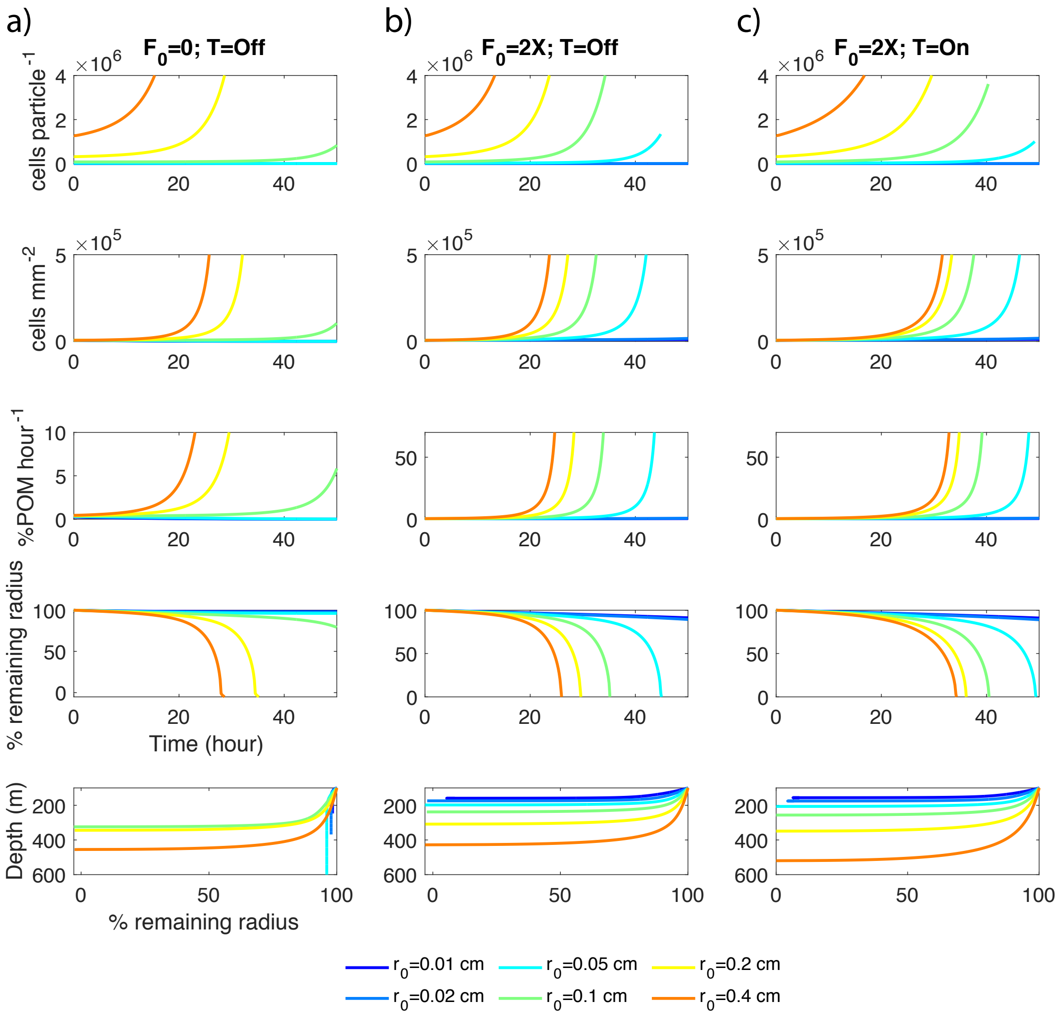

Supplementary Figure 7. Impact of temperature and encounter rate on particle organic matter (POM) degradation rates. To test the interaction between temperature and encounter rate on particle degradation rates three sets of model simulations were conducted: (a) No exchange between particles and free-living bacteria (F_i=0) and no temperature impact on bacterial growth rate (T=off), (b) Low exchange between the free-living bacterial community and the particle (F_i=2X, or ~10 cells mm^{-3} at 100 m) and no temperature impact on bacterial growth rate (T=off), (c) Low exchange between the free-living bacterial community and the particle (F_i=2X) and including the impact of temperature on microbial growth rates.

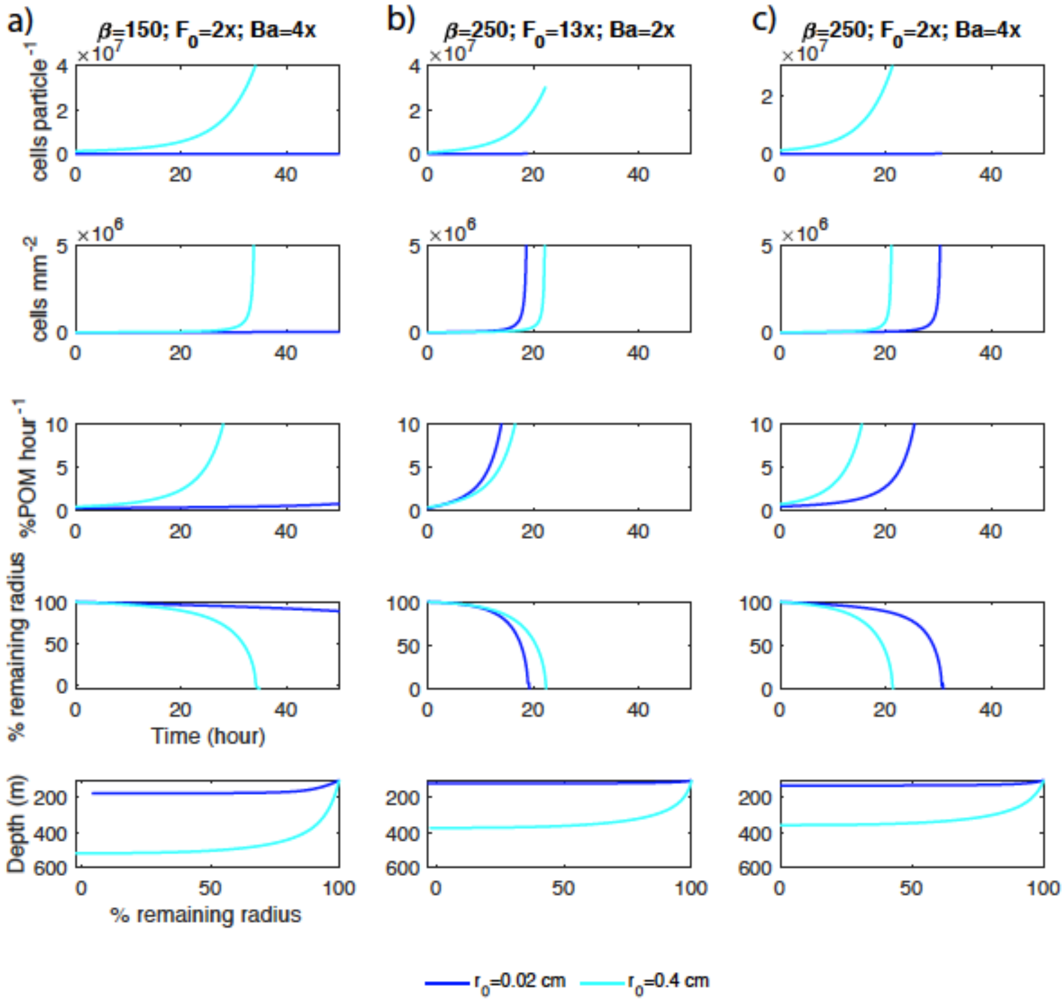

Supplementary Figure 8. Impact of particle lability, initial population density, and encounter rate on particle organic matter (POM) degradation rates. We compare the degradation dynamics of two particles with identical biological properties but different sizes (r=0.02 cm and r=0.4cm). While large particles often degrade more rapidly than similar small particles due to differences in carbon content, shifts in lability, initial density and/or encounter rate can reverse this relationship and small particles can be degraded more rapidly (middle column).

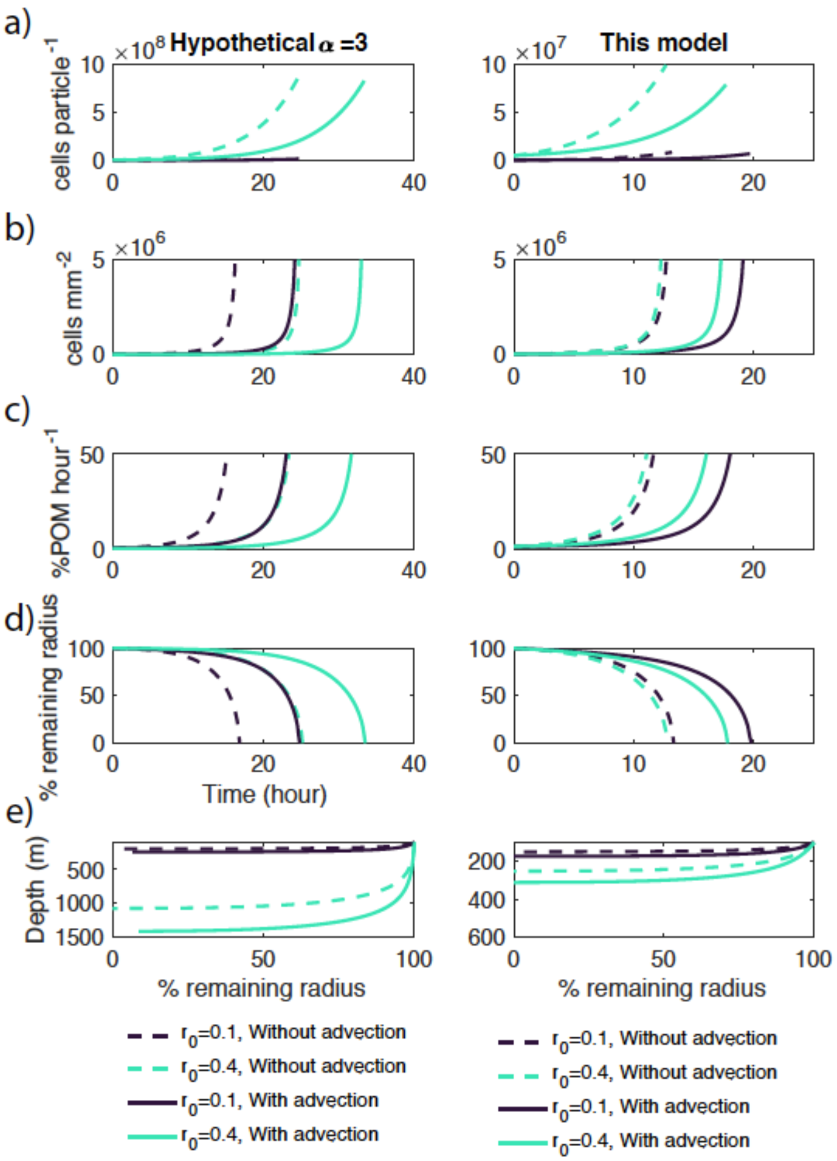

Supplementary Figure 9. Particle organic matter (POM) degradation dynamics. Here we show the change in particle-associated biomass of the primary degrader B_i (cells particle^{-1}) (a), bacterial density (cells m^{-2}) (b), particle radius (c), and particle carbon content (d) over time and (e) over depth. Results in (c) are plotted as the particle radius relative to the original particle radius (%). In all plots, the colors denote different initial particles sizes. Two model formulations are compared: a model where particles are represented as solid sinking spheres ($D_{fractal}$ =3, left column) and the default model used in the main text where particle density ranges from small dense spheres to ‘fluffy’ aggregates (right column). When large particles are not represented as aggregates, they have higher carbon content so degrade more slowly and are denser and so sink faster. We also compare both model formulations run with and without the effect of advection (solid and dashed curves, respectively). Particles degrade much faster without advection as the loss rate of LMWOM is lower and so the bacterial population is able to grow faster.

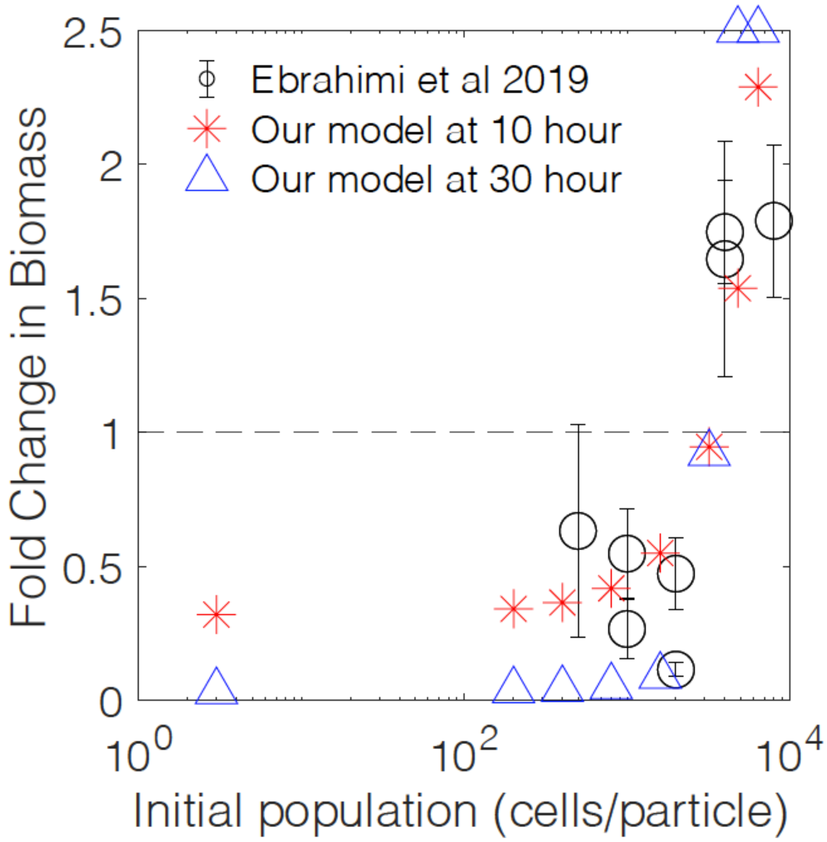

Supplementary Figure 10. Density-dependent growth validation. The model captures observed density-dependent growth of particle-associated communities. Fold change in bacterial population biomass after 10 hours of growth is shown for experimental data (Ebrahimi *et al* (2019)) and model simulations after 10 hours (red asterisks) and 30 hours (open triangles) of growth with comparable particle size and initial population. Data at 30 hours show that all the populations that are below the density threshold at initial conditions (represented here as populations of fewer than 8,000 cells per particle of 100 μm) result in true extinction on the particle. For this test, we assume no additional encounters occur after particle formation. If encounter dynamics were included, we would not see true extinction on the particles. Error bars for the open circles represent standard error of measurement from at least three measurements of particle colonization density from Ebrahimi *et al* (2019).

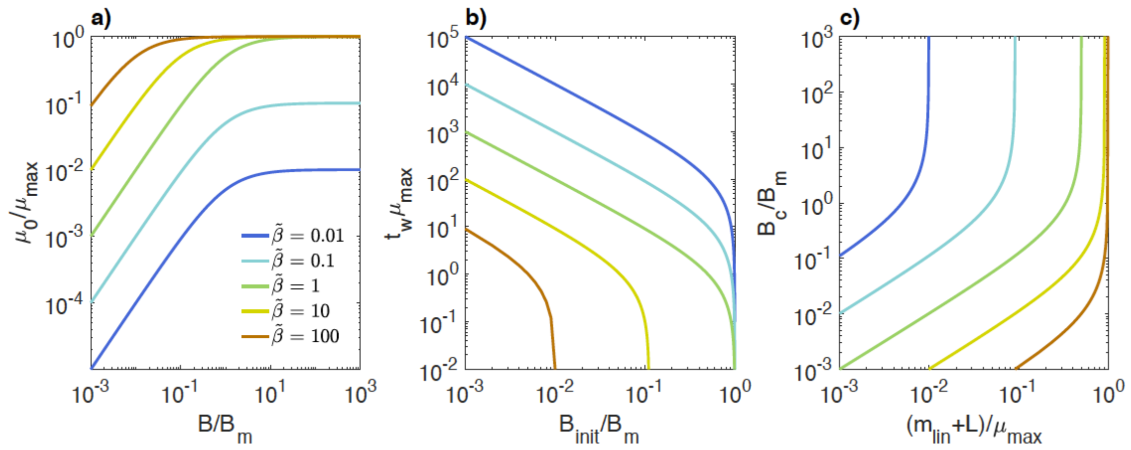

Supplementary Figure 11. Density-dependent growth of bacteria on a POC surface. Here we plot results derived from the solution to the equations presented in Supplementary Material 4. **(a)** Numerical solution for the density-dependent per capita growth rate $\mu_0 \equiv \mu\big(C_0(B)\big)$ using eqs. s28 and s33, plotted against B, the number of bacteria on the surface of POC. The lines in all panels are colored according to the lumped (and dimensionless) parameter $\tilde{\beta} = \beta y_{lwmom} / \mu_{max}$ (legend shown in panel (a) where μ_{max} is the maximum growth rate on LMWOM, and $\tilde{\beta}$ describes the ratio of the nutrient synthesis rate and the maximum uptake rate. $B_m = 4\pi R \cdot D y_{lmwom} k_m/\mu_{max}$ is another key lumped parameter that gives the characteristic scale of cell density for different bacterial and physical parameters. See Supplementary Material 4 for the precise definition of each bacterial and physical parameter. **(b)** Estimate of the waiting time t_w for different values of B_{init}, the initial number of cells on the surface of POC, by direct numerical solution of eq. s42. Here t_w is taken to be the time that the instantaneous growth rate $\frac{d}{dt} ln\, B(t)$ reaches 50% of the saturated growth rate $\mu_0(B \to \infty)$. **(c)** Estimate of the critical population size threshold B_c based on eq. s47 for various values of $\tilde{\beta}$.

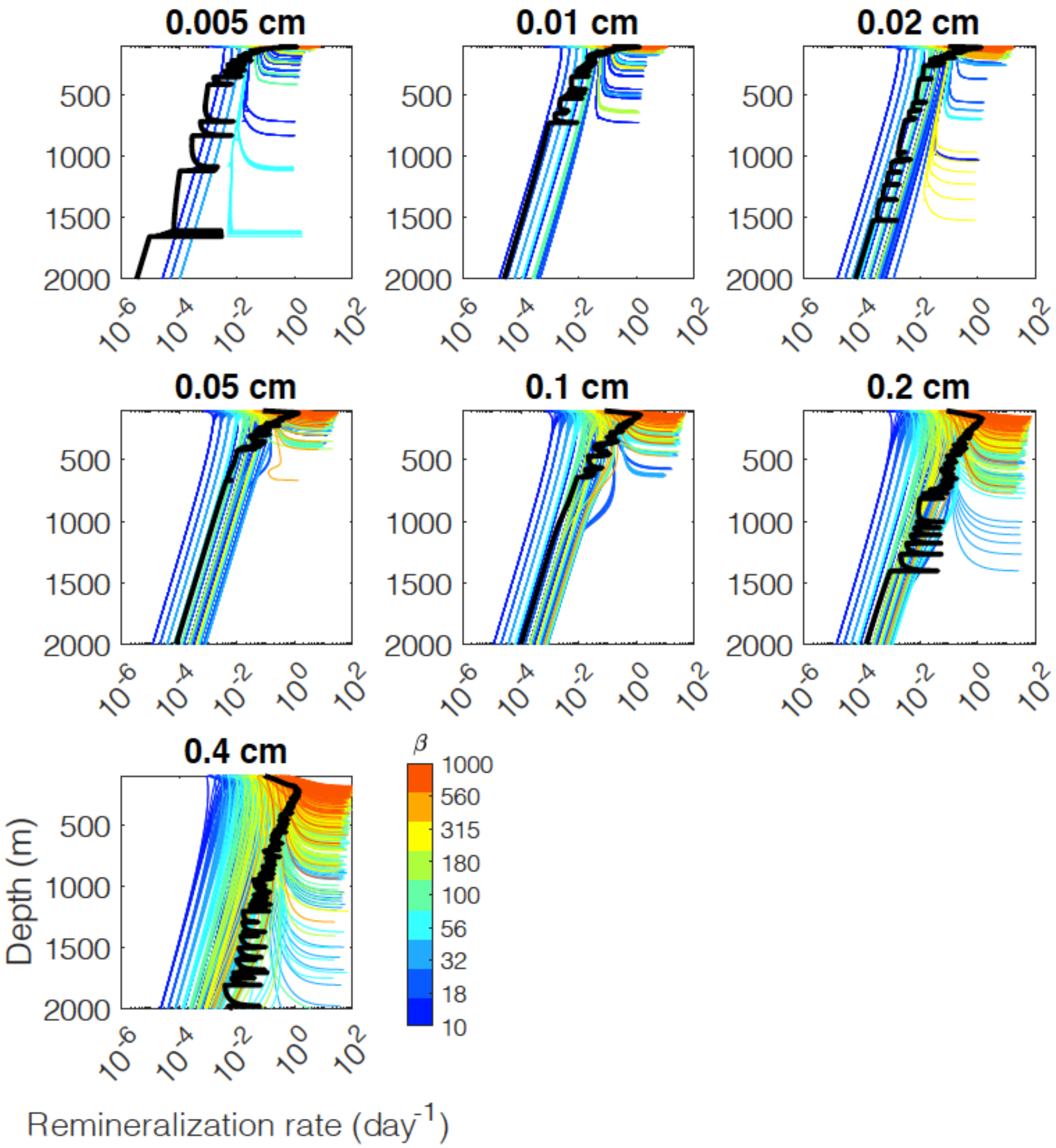

Supplementary Figure 12. Remineralization rates as a function of particle sizes, labilities, and depth. The rate of particulate organic carbon remineralization for a single particle size varies significantly as a function of the particle-associated microbial dynamics (shown by diverse thin colored curves in each subplot). The color of the thin curves corresponds to the particle lability (β). The average rate of remineralization varies over depth (thick black curves) and across particle size classes (between subplots).

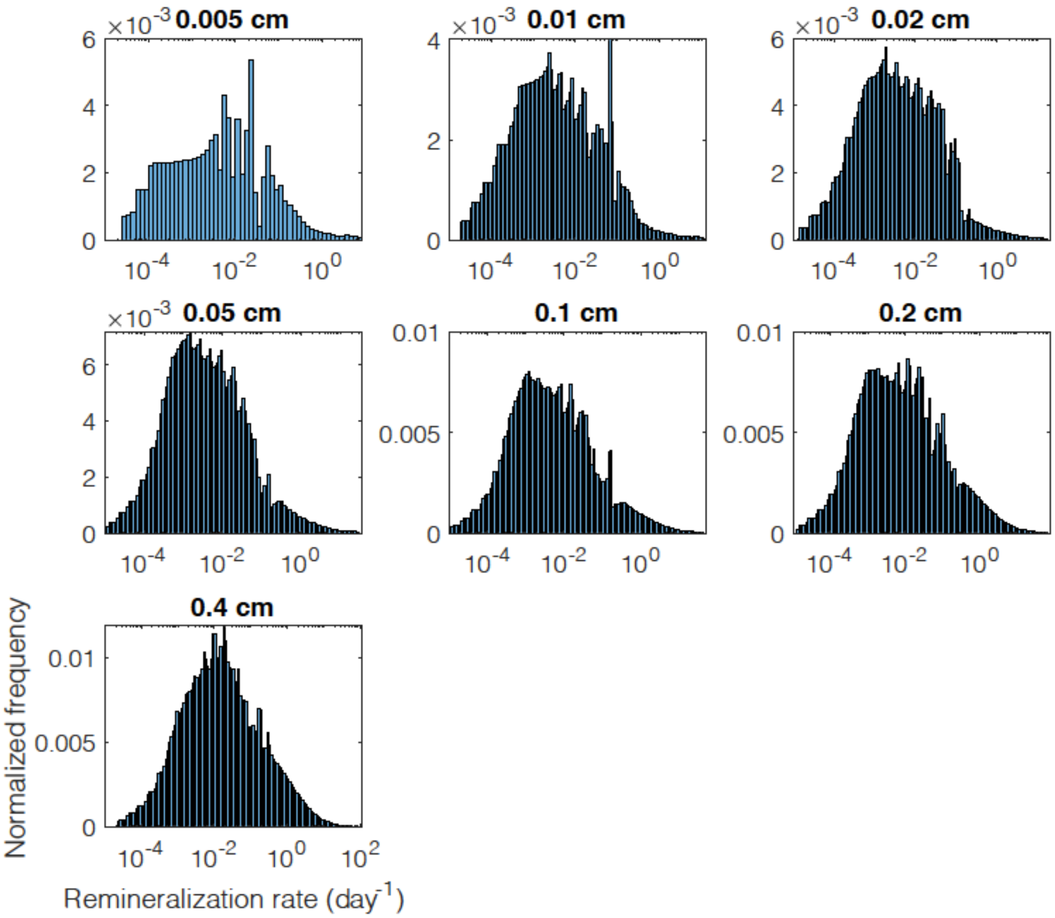

Supplementary Figure 13. Distribution of remineralization rate across particles. The distributions of particulate organic carbon remineralization rates over the upper 2,000 m for each initial particle size (n=648 particles per plot) are plotted as histograms.

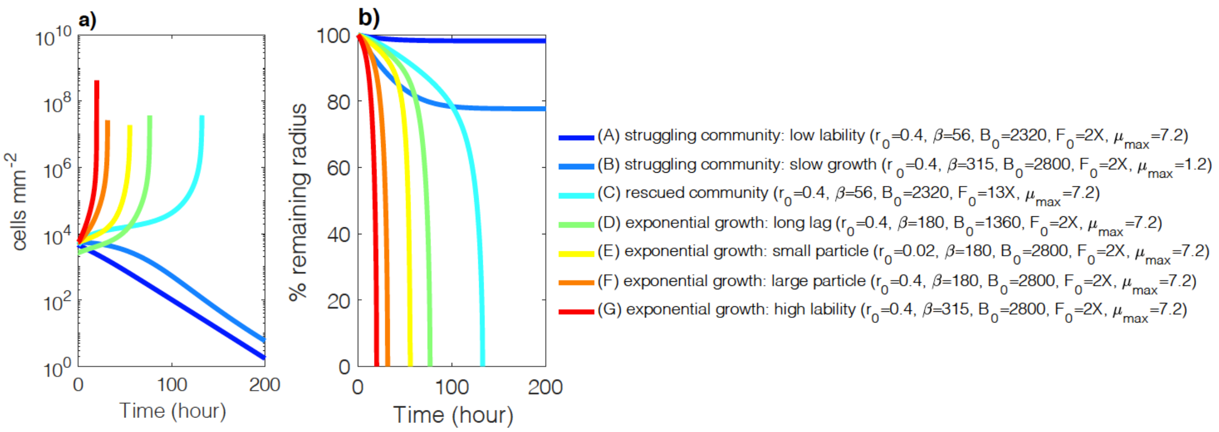

Supplementary Figure 14. Particle-associated microbial growth dynamics. Extended y-axis of main text Figure 3a plotted on a log-scale. Example particle organic carbon (POC) degradation dynamics are shown for rapidly growing populations (curves E, F, G), 'rescued' populations (C, D), and 'struggling' populations (A, B). For each simulation, the change in bacterial density on the particle surface over time (**a**), percentage changes in particle radius over time **(b)**. Model parameters for each curve are given in the legend where r_0 is the initial particle radius (cm), β is particle lability (mmol C_{POC} mmol C_{cell}^{-1} day^{-1}), B_0 is initial population density on the particle (cells mm^{-2}), F_0 is the abundance of the free-living bacterial community of the primary degrader at 100 m (where 1X is equivalent to 5 free-living cells mm^{-3}), and maximum bacterial growth rate (μ_{max}, day^{-1}).

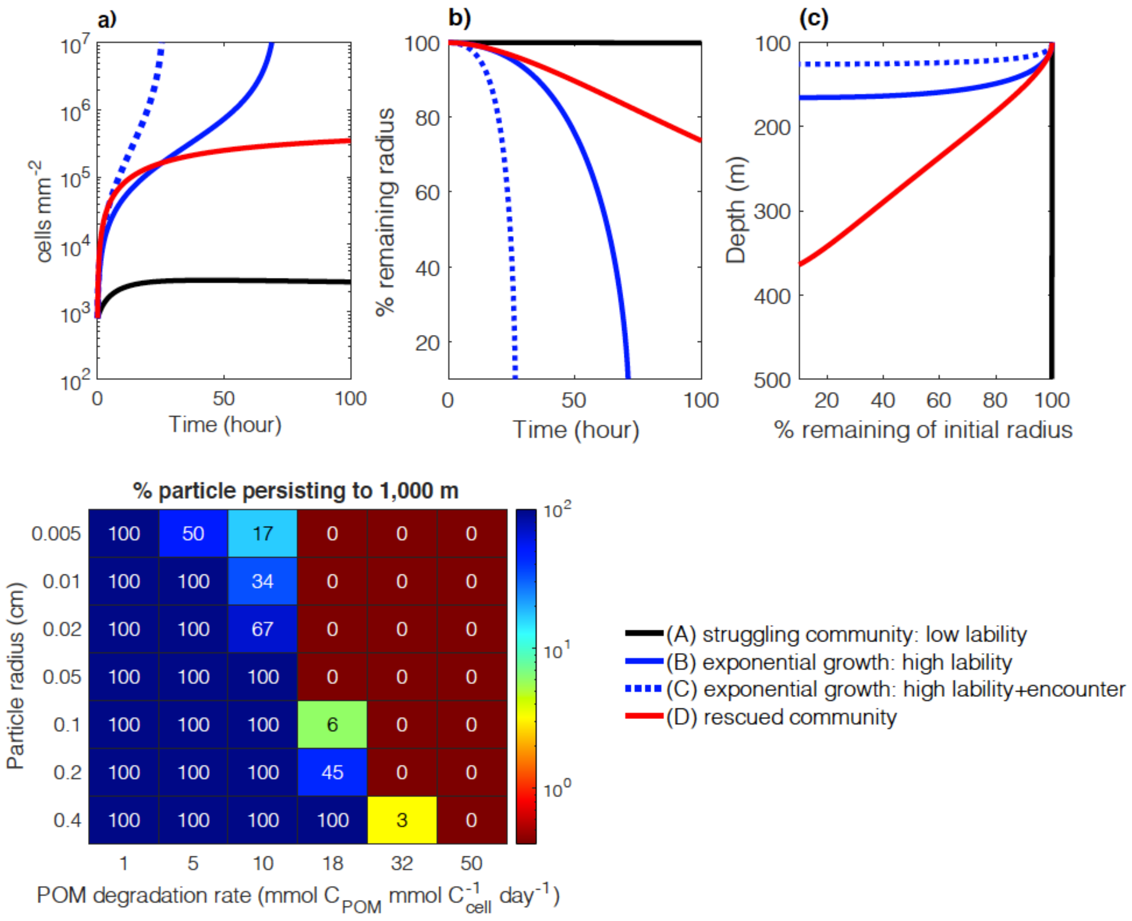

Supplementary Figure 15. Particle-associated microbial growth dynamics for direct-uptake simulations. Example degradation dynamics are shown for rapidly growing populations (curves B, C), 'rescued' populations (D), and 'struggling' populations (A). For each simulation, the change in bacterial density on the particle surface over time **(a)**, percentage changes in particle radius over time **(b)** and depth **(c)** are shown. To quantify the impact of microbial growth dynamics on particle organic matter (POM) persistence at depth, we estimate the fraction of particles within a given lability class and radius at formation that persist below 1,000 m **(d).** The colors and values in each grid correspond to the percentage of parameter combinations for model parameters other than lability and initial radius (n=72) that persist past 1,000 m depth (see Supplementary Material 1).

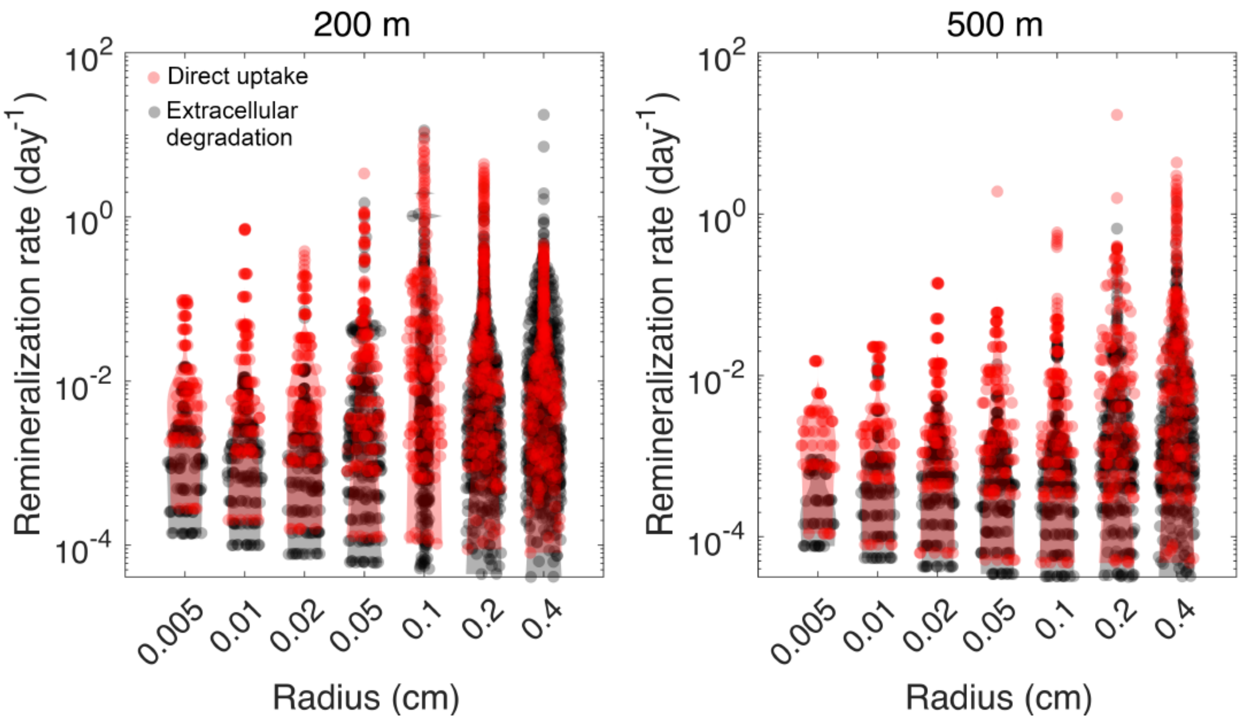

Supplementary Figure 16. Remineralization rates for model simulations with direct-uptake (red) and extracellular degradation (black) at 200 and 500 m across different particle size classes.

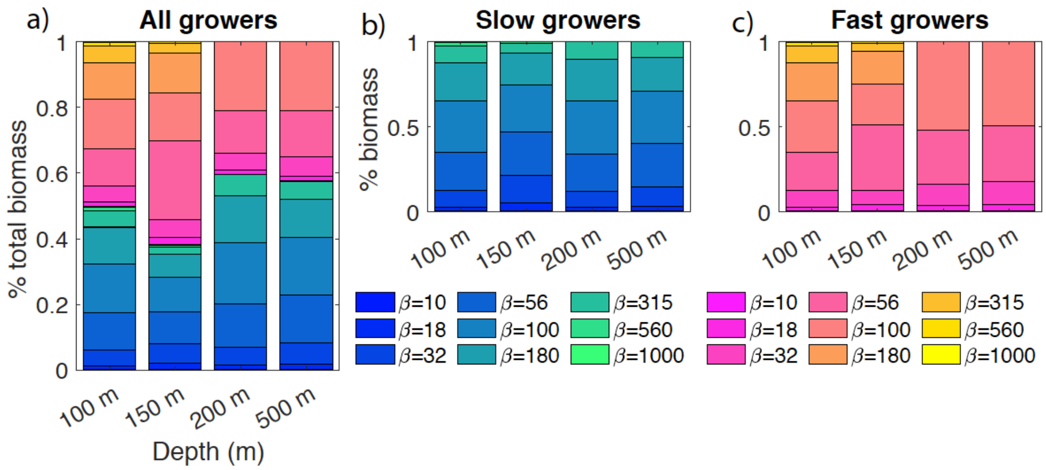

Supplementary Figure 17. Shifts in bacterial community composition with depth. Bacterial populations are grouped based on their interaction with particle organic carbon (POC) (represented as particle lability or β) and their maximal growth rate (fast growers are bacteria with μ_{max}=7.2 day^{-1}, slow growers are bacteria with μ_{max}=1.2 day^{-1}). **(a)** represents relative abundance of all particle-associated bacterial populations at formation (100 m), 150 m, 200 m and 500 m. **(b-c)** represent relative abundance within different categorical groups of slow growers and fast growers, respectively. Of note, this is not a prediction of how observed bacterial growth rates (μ_i) will vary over depth (growth rates are also a function of temperature and LMWOM concentration) but rather how bacterial diversity might vary.

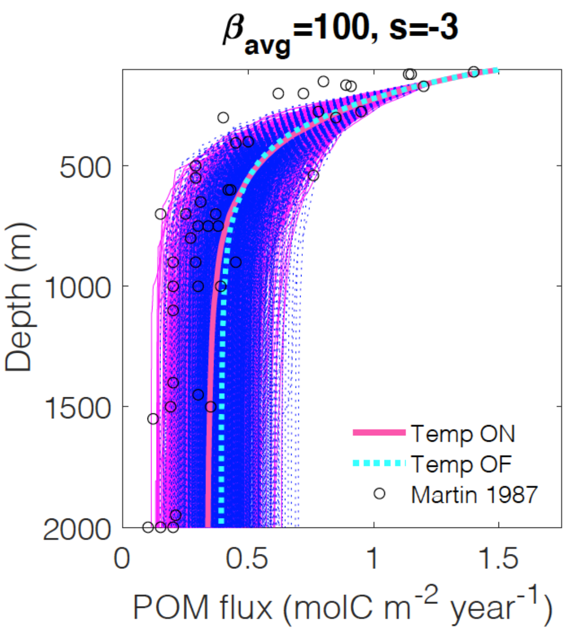

Supplementary Figure 18. Impact of temperature on water column particle organic matter (POM) flux. Total POM flux over depth for 1,000 stochastic simulations is shown. Each pink (temperature dependent growth and mortality simulations, "Temperature on") and blue (temperature independent growth and mortality simulations, "Temperature off") thin curve is a single model experiment and is the sum of 69,000 randomly initialized particles (with particle size spectra power law exponent *s*=-3 and lognormal distributed lability with $\beta_{average}$= 100 mmol C_{pom} mmol C_{cell}^{-1}). The thicker pink and blue curves represent the average of the 1,000 thin curves, respectively for Temperature on and off simulations. Open circles represent observed POM fluxes from the North Pacific[24].

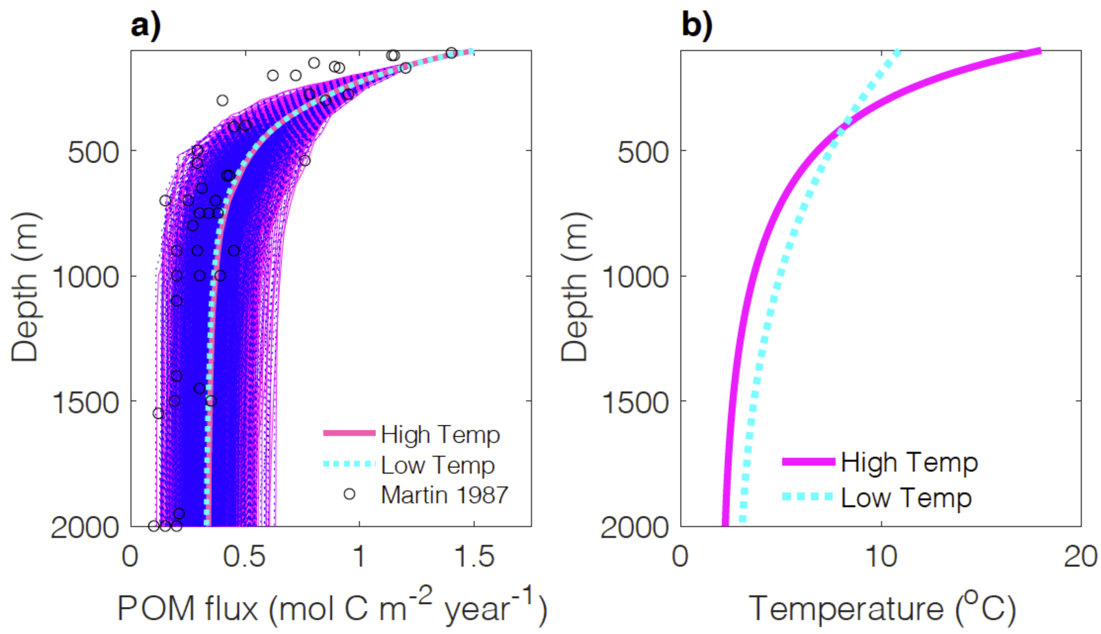

Supplementary Figure 19. Impact of regional differences in temperature on particle organic matter (POM) flux. The 'low temperature' profile has a narrow temperature gradient from 12ºC to 3ºC (0-2,000 m), based on the temperature profile from the subpolar North Atlantic region (data from CCHDO Hydrographic Cruise 64PE20000926). The default temperature profile ('High Temp') captures a large change of temperature over the water column (26ºC to 2ºC) from 0-2,000 m. Panel a) shows the results from 1,000 stochastic simulations conducted using the low temperature profile (pink line) and 1,000 simulations conducted using the high temperature profile (blue dashed lines). Observed POM flux measurements from Martin *et al* (1987) are plotted for reference (as open circles). Panel b) shows the two temperature profiles for the upper 2,000m.

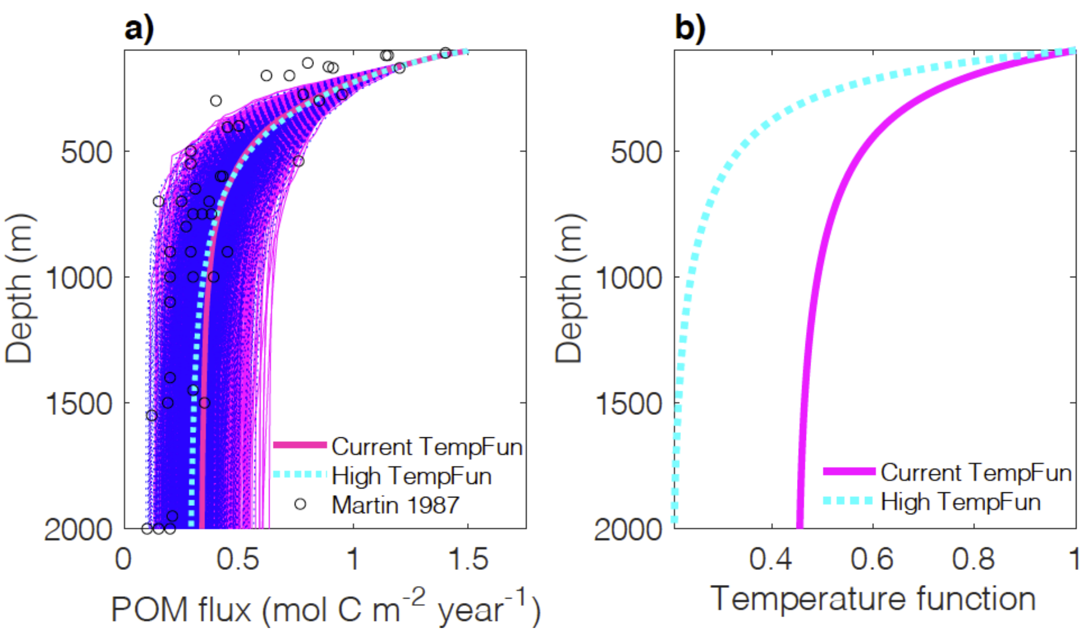

Supplementary Figure 20. Impact of larger temperature-associated decrease in particle organic matter (POM) flux. Both simulations are run with the same temperature profile as described by eq. s10, however, vary in their associated temperature limitation on growth and mortality (eq. s9). The high temperature function (dashed blue curves) represents a larger temperature-associated decrease in growth rate such that growth rate decreases by 80% in the upper 2,000 m, specifically: $\gamma_{T,z} = \tau e^{\left[2A_E\left(\frac{1}{T_z+273.15}-\frac{1}{T_0}\right)\right]}$. The current temperature function (solid pink curves) represents the temperature function described by eq. s9: $\gamma_{T,z} = \tau e^{\left[A_E\left(\frac{1}{T_z+273.15}-\frac{1}{T_0}\right)\right]}$. Panel a) shows the results from 1,000 stochastic simulations conducted using the normal temperature function (pink line) and 1,000 simulations conducted using the high temperature function (blue dashed lines). Observed POM flux measurements from Martin *et al* (1987) are plotted for reference (as open circles). Panel b) shows the two temperature function profiles for the upper 2,000m.

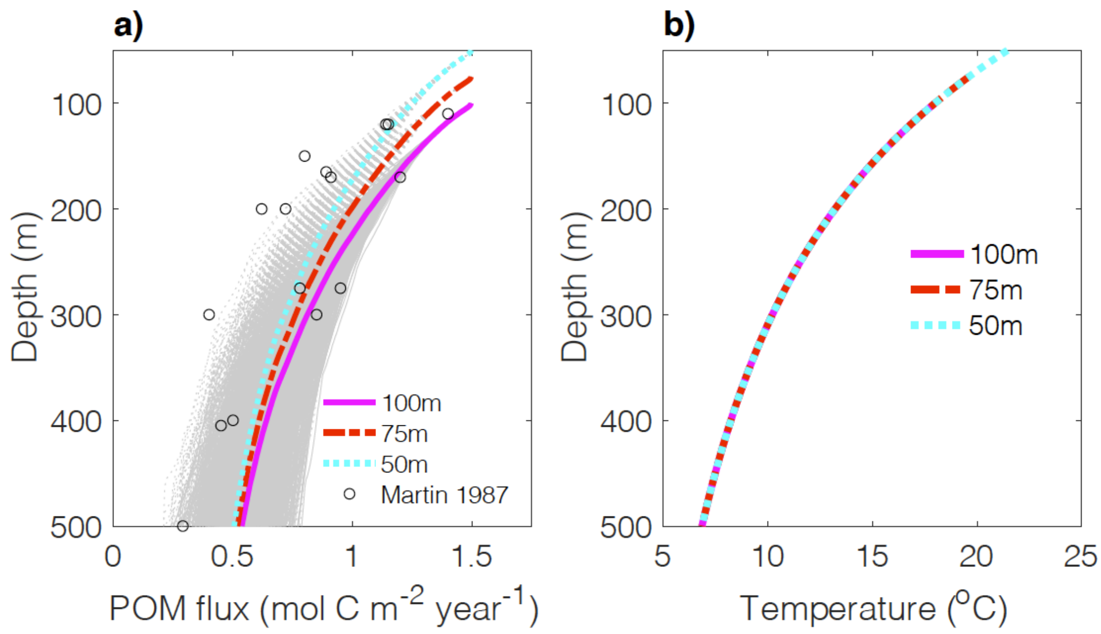

Supplementary Figure 21. Sensitivity of particle formation depths to large-scale particle organic matter (POM) fluxes. Panel a) shows the shifts in POM fluxes over depth that result from varying the depth of particle formation (50, 75, and 100 m). Each gray line is the integrated flux for 69,000 stochastically initialized particles (particle size spectra *s*=-3). For each parameter set, 1,000 stochastic simulations were conducted (1,000 gray lines) and the average is shown in the thick colored line. The data from Martin *et al* (1987) (in open circles) are also shown. Panel b) shows the temperature profile for the upper 500 m. Particle formation depth impacts encounter dynamics and the temperature function. These results demonstrate how variable particle formation depths can significantly impact the POM flux in the surface ocean (< 500 m). However, we see convergence in the model results below ~500 m.

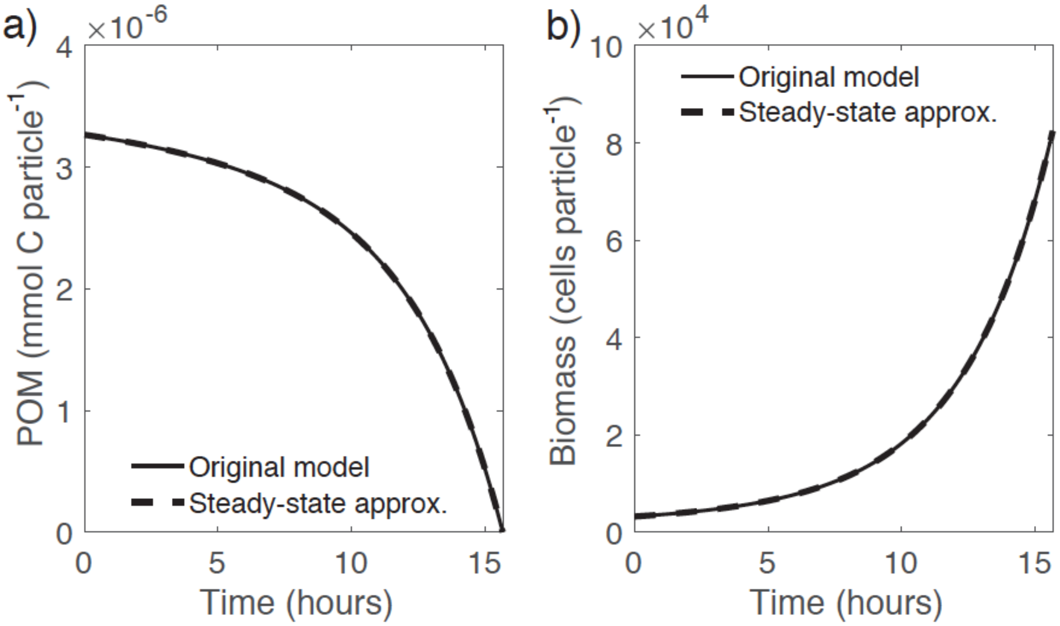

Supplementary Figure 22. Comparison between steady state approximation for low molecular organic matter (LMWOM) concentration and simulations that explicitly resolve diffusion of LMWOM away from a particle. Particle organic matter (POM) concentration **(a)** and particle-associated biomass **(b)** over time of the model simulation are shown. See Supplementary Material 2 for details.

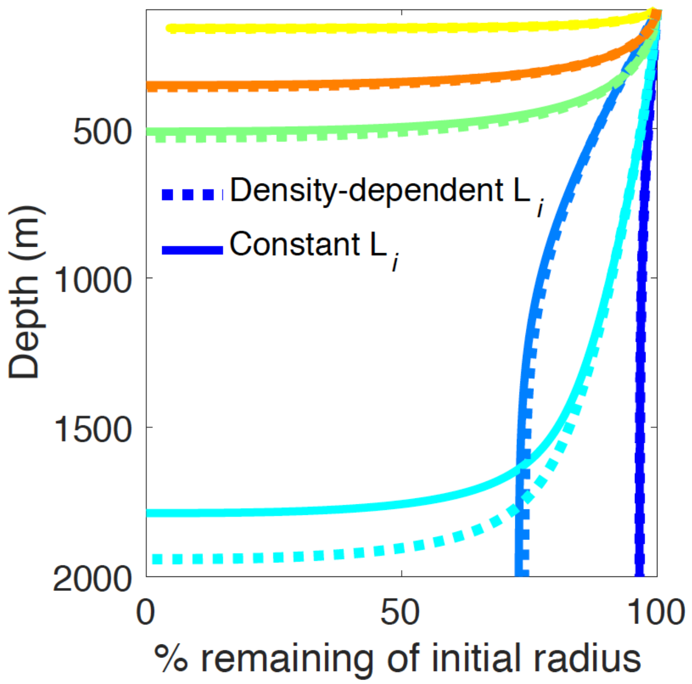

Supplementary Figure 23. Sensitivity of model dynamics to bacterial detachment formulation. Model simulations with the default constant detachment rates (solid lines) are compared against a density-dependent detachment formulation (dashed lines). The x-axis represents the fraction of the particle radius remaining. The choice of detachment formulation does not qualitatively change the model dynamics (Supplementary Material 2).

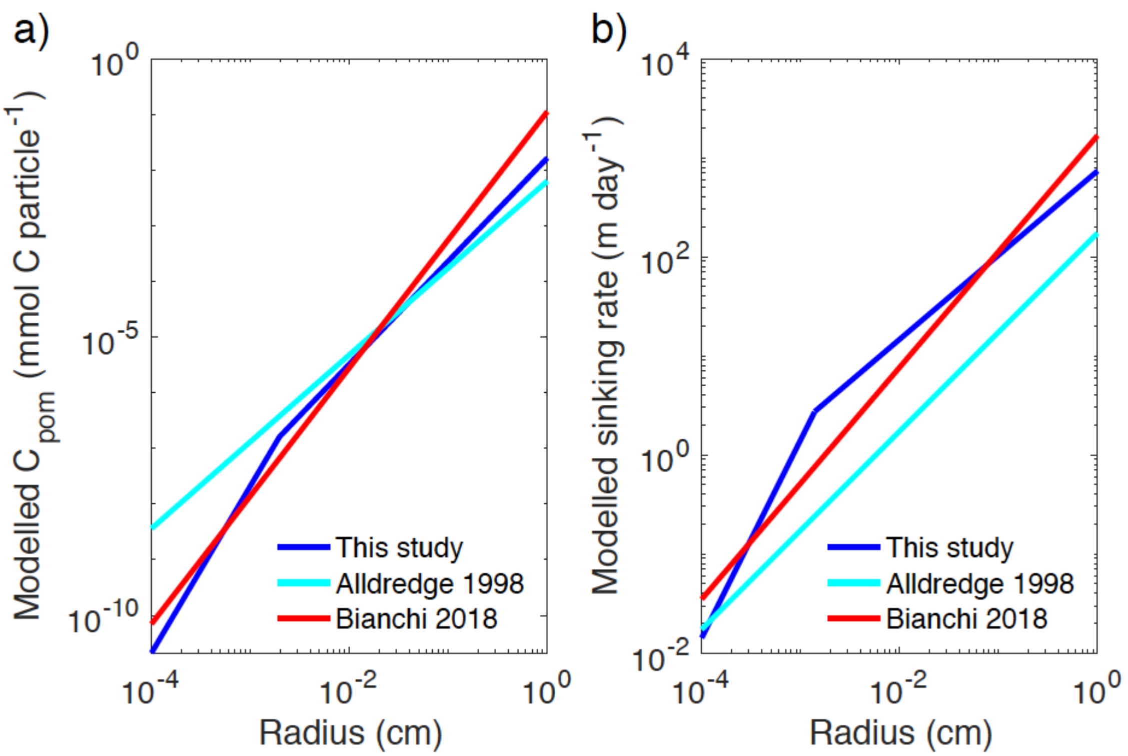

Supplementary Figure 24. Comparison of different particle density and particle sinking relationships. The relationship between particle radius r_i and particle organic matter (POM) concentration $C_{pom,i}$ **(a)**, and sinking rate ω_i **(b)** are shown for the formulation used in this paper (after Omand *et al* 2020) and the fitted equations of Alldredge *et al* (1998) and Bianchi *et al* (2018). Despite differences in carbon content and sinking speeds, our mechanistic model based on Omand *et al* 2020 shows quantitatively similar results to Alldredge *et al* (1998) (see Supplementary Material 2 and Supplementary Figure 25).

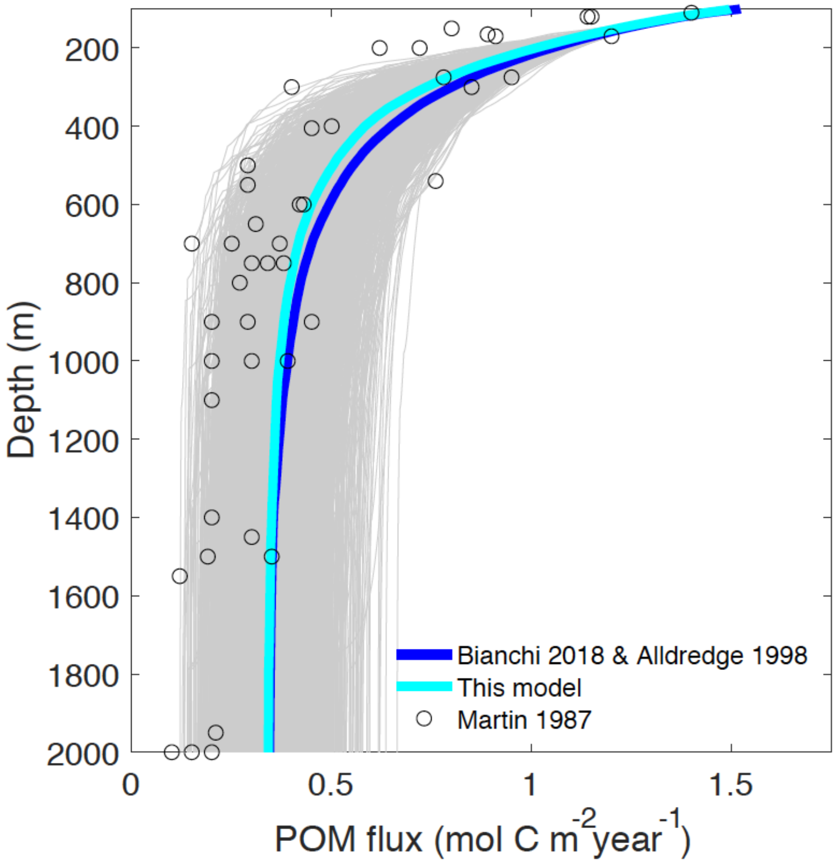

Supplementary Figure 25. Impact of particle density and sinking dynamics on modeled large-scale particle organic matter (POM) fluxes. The integrated POM flux for two model simulations is compared: the model presented in the main text and a model using empirically derived relationship between particle size, density and sinking speeds (Bianchi *et al* 2018 and Alldredge *et al* 1998). For each parameter set, 1,000 stochastic simulations were conducted (1,000 gray lines) and the average is shown as the thick colored line. The data from Martin *et al* (1987) (in open circles) are also shown.

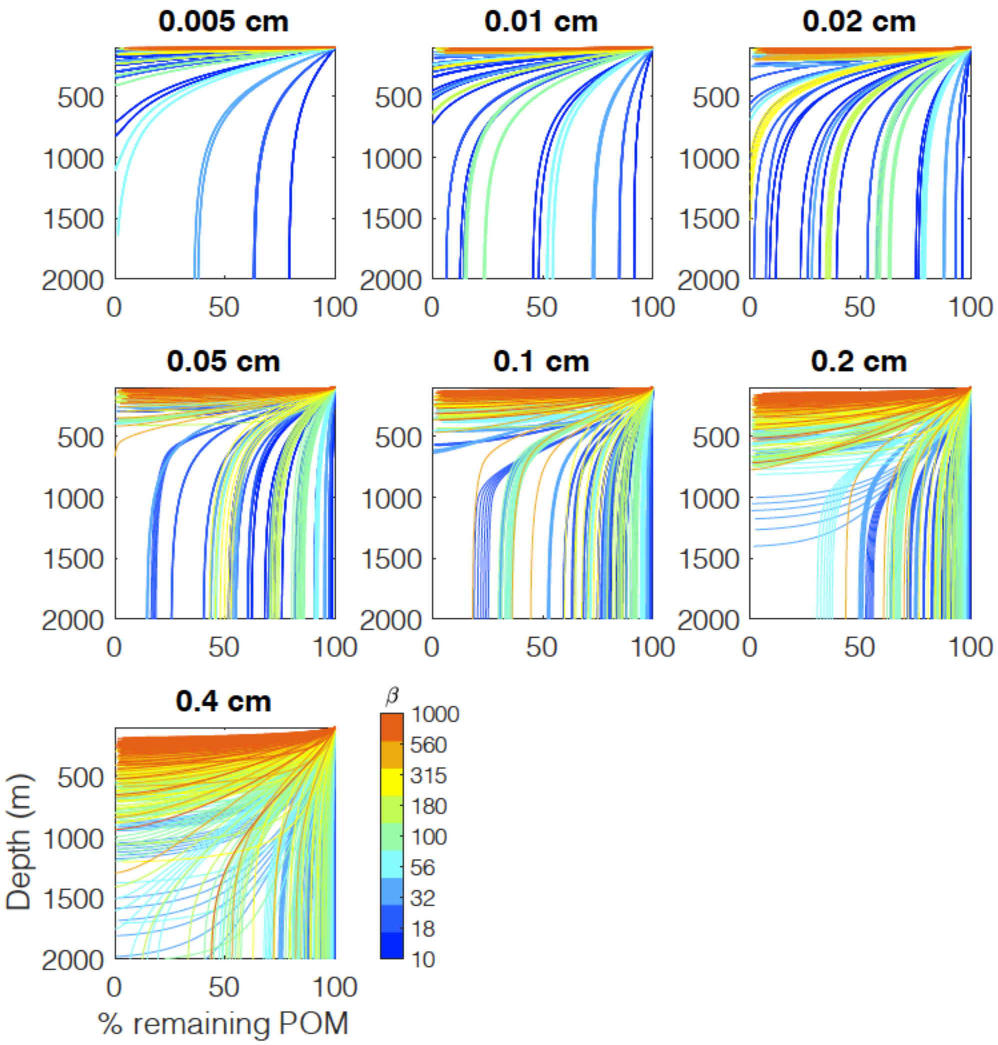

Supplementary Figure 26. Particle organic matter (POM) degradation profiles over depth (n=4,536). Values on the x-axis represents the percentage of the remaining POM content in each particle. The 7 subplots represent the 7 initial particle radiuses used in our study. The colors of the curves represent the lability of each particle (β, mmol C_{pom} mmol C_{cell}^{-1} day^{-1}) with hotter colors corresponding to higher lability.

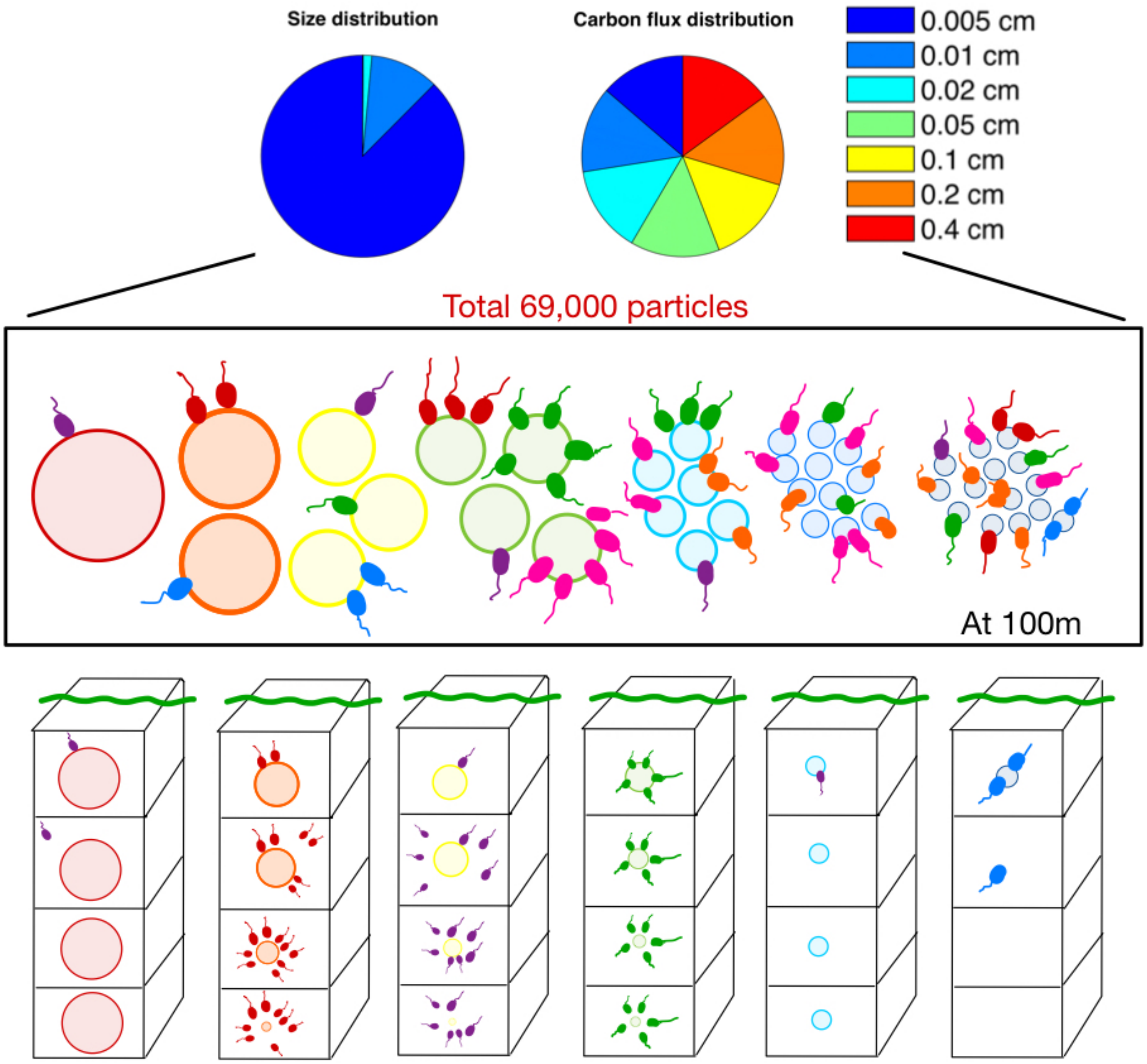

Supplementary Figure 27. Schematic illustration of the water column model. The number of particles at formation (100 m) is defined based on observed particle size spectra in the ocean, here illustrated for power law exponent *s*= -3. Each particle is randomly assigned with an initial radius, lability (log-normal distribution), and a set of biological parameter values (maximal growth rate, initial biomass, and free-living bacterial abundance of group Bi) using a uniform distribution. The dynamics of each particle is then resolved independently as it sinks through the water column. The results are summed to obtain the total particle organic carbon (POC) flux throughout the water column.

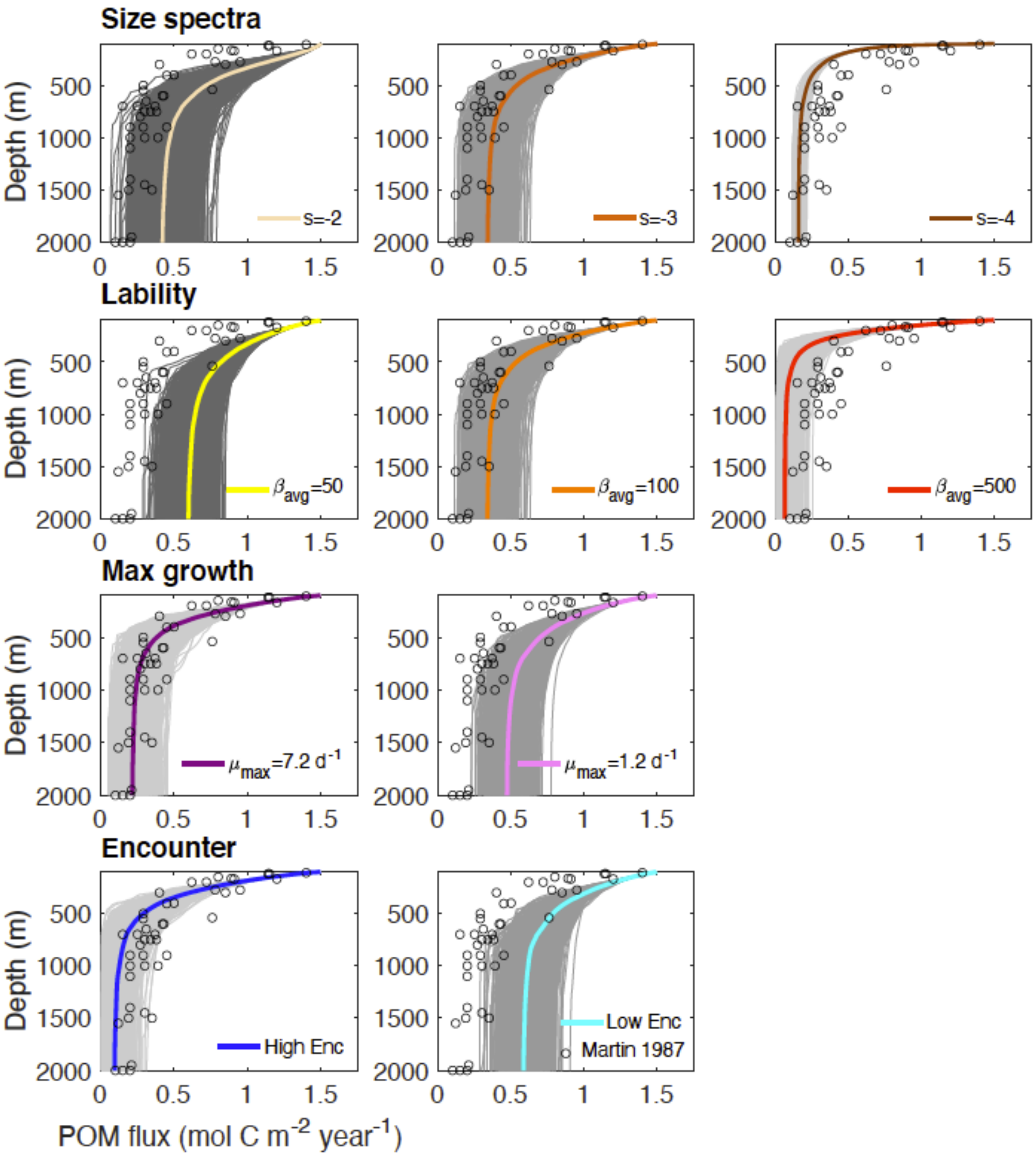

Supplementary Figure 28. Microbial control on large-scale particle organic matter (POM) fluxes (Extended main text Figure 4**).** Shifts in POM fluxes over depth are shown as a function of varying particle and microbial dynamics. Each gray line is the integrated flux over 2,256 to 763,615 stochastically initialized particles (depending on particle size spectra). For each parameter set, 1,000 stochastic simulations were conducted (1,000 gray lines of different shades) and the average is shown as the thick colored line. The data from Martin *et al* (1987) (in open circles) are also shown. POM transfer efficiency decreases (larger attenuation exponent *b*) with more negative particle size spectra power law exponent *s*, higher mean particle lability (β_{avg} in mmol C_{POM} mmol C_{cell}^{-1} day^{-1}), higher maximal growth rate (μ_{max} in day^{-1}), and higher particle-microbe encounter rate (Enc).

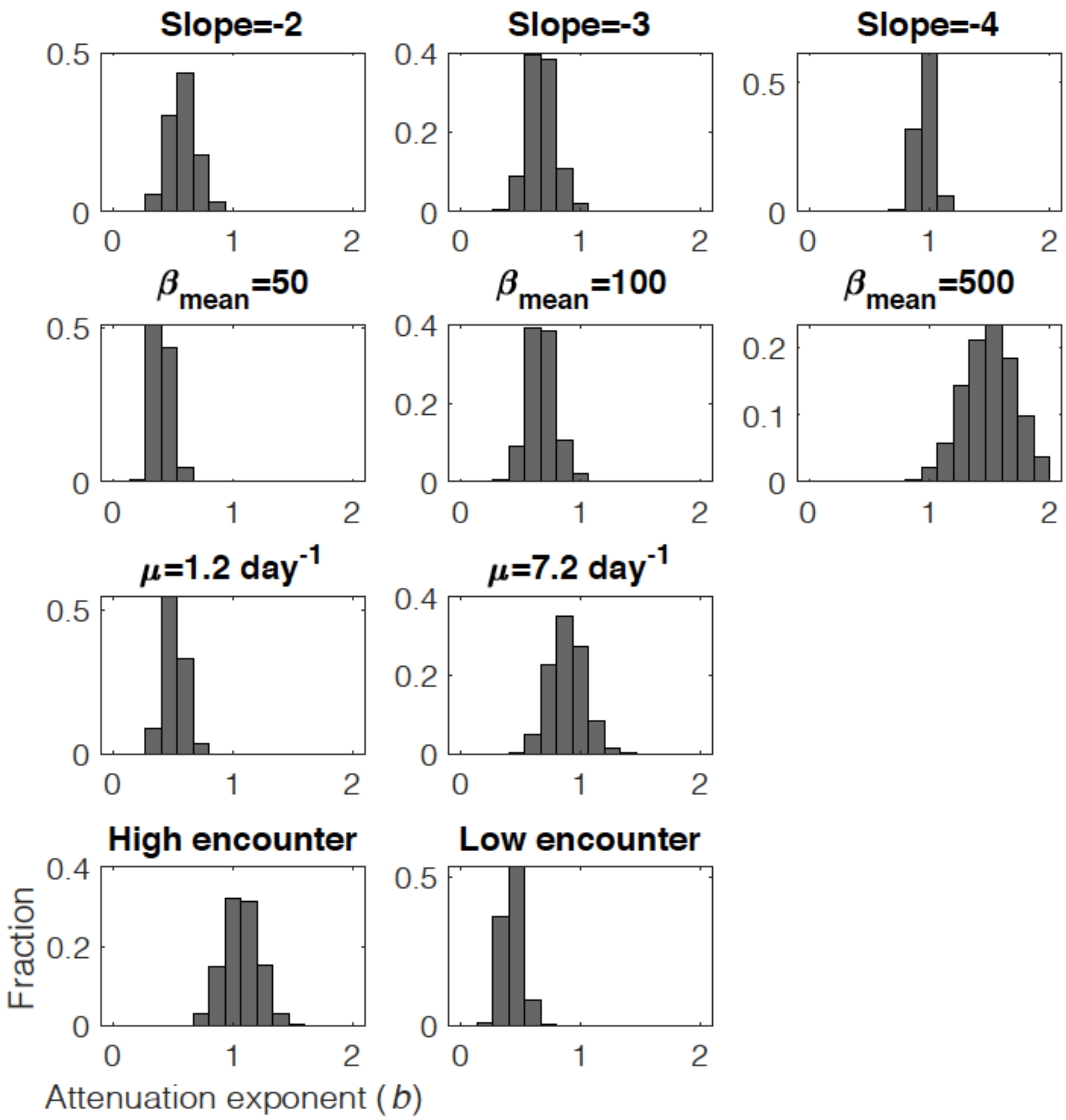

Supplementary Figure 29. Distribution of modeled attenuation exponents (*b*) for model simulations shown in main text Figure 4

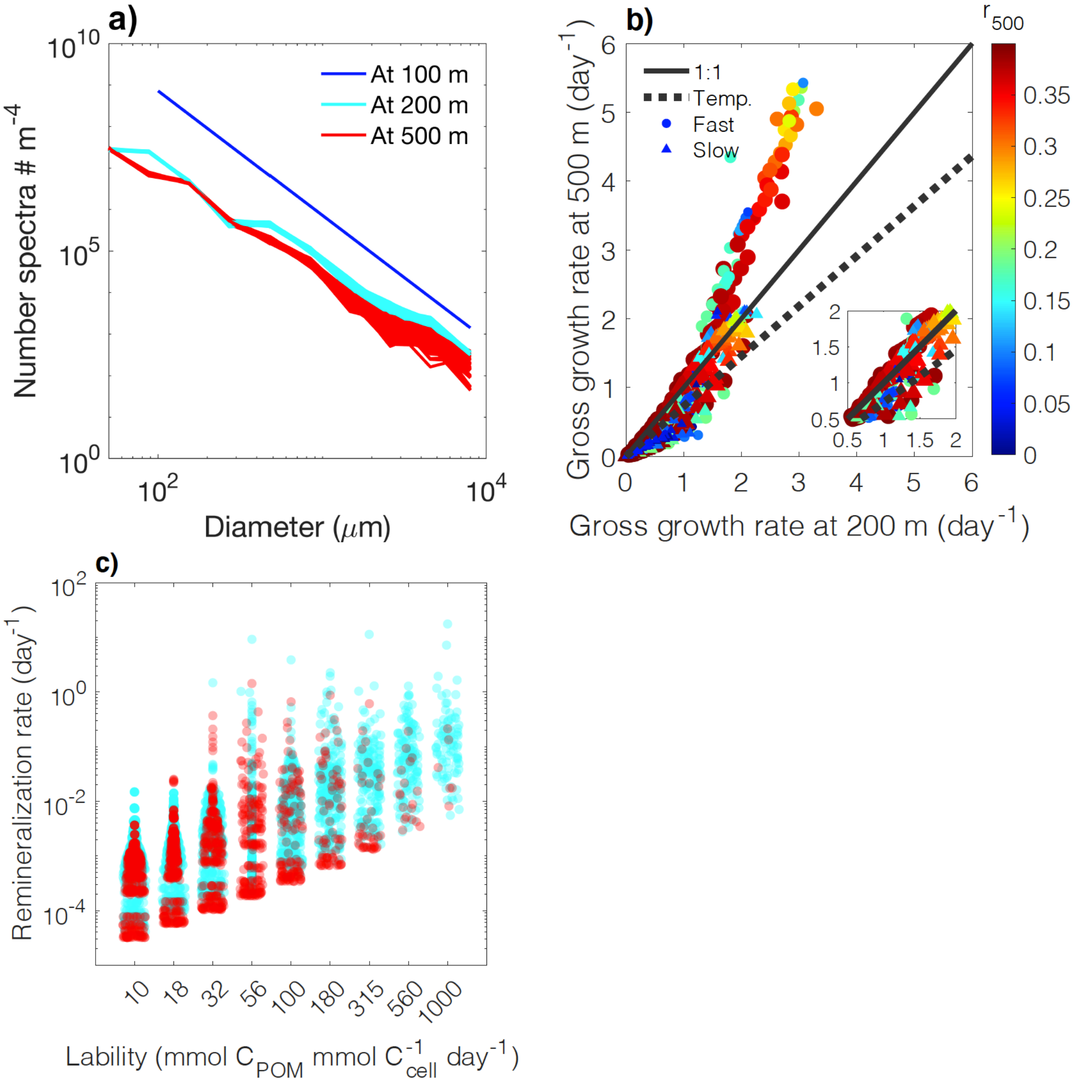

Supplementary Figure 30. Modeled bacterial dynamics on particles. Panel (a) shows the particle size spectra at 100 m (blue), 200 m (light blue), and 500 m (red) for 1,000 stochastic model simulations initialized with a particle size spectra (PSD) slope of -3. PSD are calculated for each depth as the concentration of particles in certain size class (C_i) divided by the range of particle diameters used to define the size class (Δd_i) ($PSD_i = \frac{C_i}{\Delta d_i}$ following Iversen *et al* 2011). **Panel (b)** plots the comparison between particle-associated bacterial growth rates at 200 m and 500 m. Symbol size and color represent the particle size at 500 m. The symbol shape denotes whether the bacterial colonizer (B_i) is a fast grower (circle) or slow grower (triangle). The dash line represents the hypothetical growth rate at 500 m if temperature was the only factor driving the change in bacterial growth rate with depth. **Panel (c)** shows the relationship between particle organic matter (POM) lability and particle remineralization rate at 200 m and 500 m for 1,000 stochastic model simulations.

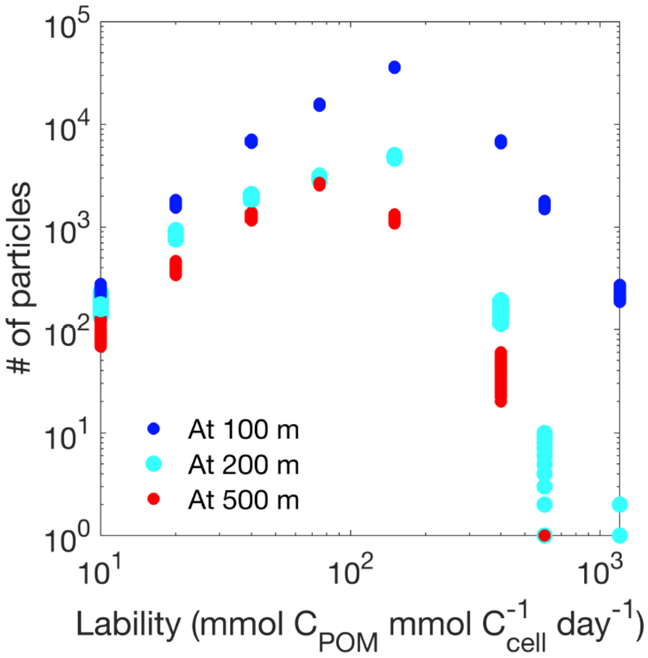

Supplementary Figure 31. Distribution of particle lability for particles at formation (100 m in dark blue), 200 m (light blue), and 500 m (red). The number of particles (summed across the size spectrum) is plotted versus lability. As the particles sink, higher lability particles are consumed more rapidly and so the lability distribution shifts with depth such that total particle organic matter (POM) on average becomes more recalcitrant. However, there is a small fraction of persistent but very labile particles that are present at depth due to the combination of model parameters stochastically assigned to these particles.

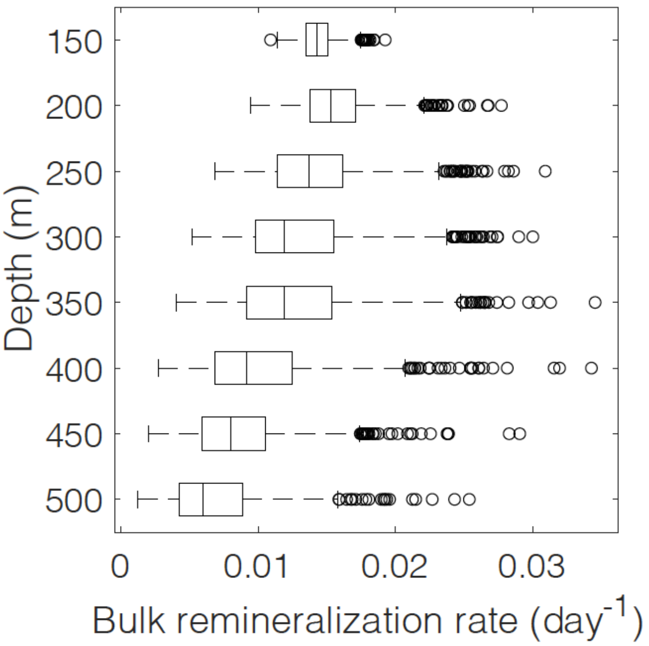

Supplementary Figure 32. Changes in bulk remineralization rates with depth. Remineralization rates of particle organic carbon (POC) over depth are calculated for each particle as ($\frac{dC_{POC,i}}{dt}\frac{1}{C_{POC,i}}$). Bulk rates are then calculated for 50 m depth bins by averaging over all particles (n=69,000 particles with particle size spectra power law exponent *s*=-3). Each box plot represents the distribution of bulk remineralization rates for a given depth across all 1,000 stochastic experiments, in which the open circles are the outliers.

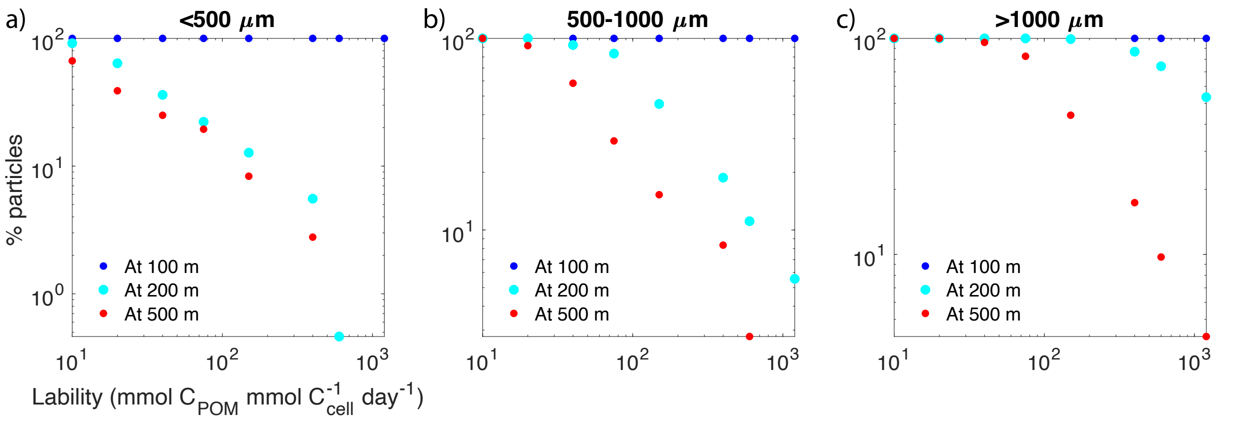

Supplementary Figure 33. Particle organic matter (POM) consumption rate over depth as a function of lability and size. The percentage of particles remaining at formation (100 m thus 100% in dark blue), 200 m (light blue), and 500 m (red) is shown as a function of lability (x-axis) and initial particle size: **(a)** particles with initial radius < 500 μm, **(b)** particles with initial radius 500-1000 μm, **(c)** particles with initial radius >1,000 μm. Due to faster sinking speeds, more large particles persist at 200 and 500 m relative to small particles.

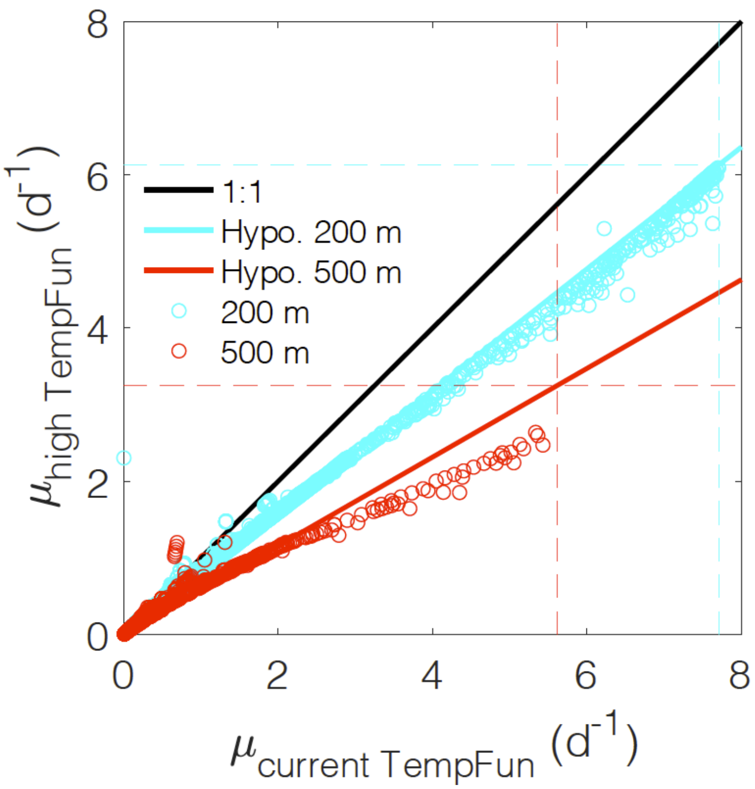

Supplementary Figure 34. Impact of larger temperature-associated decrease in heterotrophic growth rates of particle-associated microorganisms. The modeled growth rates of particle-associated microorganisms using the current temperature function (x-axis) and higher temperature function (y-axis) are compared at 200 m (in cyan) and 500 m (in red). The cyan and red line represents the hypothetical growth rate at 200 m and 500 m respectively if the difference in temperature functions was the only factor driving the change in microbial growth rate with depth. The black line is the 1:1 line. The dashed lines indicate the maximum growth rate (i.e., no limitation by LMWOM). See Supplementary Figure 20 for a comparison of the POC fluxes between these two simulations.

Supplementary References

1. Mislan, K. A. S., Stock, C. A., Dunne, J. P. & Sarmiento, J. L. Group behavior among model bacteria influences particulate carbon remineralization depths. *Journal of Marine Research* **72**, 183-218(36) (2014).
2. Ebrahimi, A., Schwartzman, J. & Cordero, O. X. Cooperation and spatial self-organization determine rate and efficiency of particulate organic matter degradation in marine bacteria. *PNAS* (2019) doi:10.1073/pnas.1908512116.
3. Stein, W. D. & Litman, T. Simple Diffusion of Nonelectrolytes and Ions. in *Channels, Carriers, and Pumps* 37–80 (Elsevier, 2015). doi:10.1016/B978-0-12-416579-3.00002-2.
4. Ploug, H., Hietanen, S. & Kuparinen, J. Diffusion and advection within and around sinking, porous diatom aggregates. *Limnol. Oceanogr.* **47**, 1129–1136 (2002).
5. Bianchi, D., Weber, T. S., Kiko, R. & Deutsch, C. Global niche of marine anaerobic metabolisms expanded by particle microenvironments. *Nature Geosci* **11**, 263–268 (2018).
6. Moradi, N. *et al.* A new mathematical model to explore microbial processes and their constraints in phytoplankton colonies and sinking marine aggregates. *Science Advances* **4**, eaat1991 (2018).
7. Kiørboe, T., Ploug, H. & Thygesen, U. H. Fluid motion and solute distribution around sinking aggregates I : Small-scale fluxes and heterogeneity of nutrients in the pelagic environment. *Marine Ecology - Progress Series* **211**, 1–13 (2001).
8. Appendix A - Thermodynamic Properties. in *Fundamentals of Salt Water Desalination* (eds. El-Dessouky, H. T. & Ettouney, H. M.) 525–563 (Elsevier Science B.V., 2002). doi:10.1016/B978-044450810-2/50013-0.
9. Liu, S. *et al.* Different carboxyl-rich alicyclic molecules proxy compounds select distinct bacterioplankton for oxidation of dissolved organic matter in the mesopelagic Sargasso Sea. *Limnology and Oceanography* **65**, 1532–1553 (2020).
10. Suttle, C. A. Marine viruses — major players in the global ecosystem. *Nat Rev Microbiol* **5**, 801–812 (2007).

11. Breitbart, M., Bonnain, C., Malki, K. & Sawaya, N. A. Phage puppet masters of the marine microbial realm. *Nat Microbiol* **3**, 754–766 (2018).
12. Dutkiewicz, S. *et al.* Capturing optically important constituents and properties in a marine biogeochemical and ecosystem model. *Biogeosciences* **12**, 4447–4481 (2015).
13. Zakem, E. J. *et al.* Ecological control of nitrite in the upper ocean. *Nature Communications* **9**, 1206 (2018).
14. Eppley, R. W. Temperature and phytoplankton growth in the sea. *Fishery Bulletin* **70**, 1063–1085 (1972).
15. Lambert, B. S., Fernandez, V. I. & Stocker, R. Motility drives bacterial encounter with particles responsible for carbon export throughout the ocean. *Limnology and Oceanography Letters* **4**, 113–118 (2019).
16. Sunagawa, S. *et al.* Structure and function of the global ocean microbiome. *Science* **348**, (2015).
17. Kiørboe, T., Grossart, H.-P., Ploug, H. & Tang, K. Mechanisms and Rates of Bacterial Colonization of Sinking Aggregates. *Appl. Environ. Microbiol.* **68**, 3996–4006 (2002).
18. Kiørboe, T., Tang, K., Grossart, H.-P. & Ploug, H. Dynamics of Microbial Communities on Marine Snow Aggregates: Colonization, Growth, Detachment, and Grazing Mortality of Attached Bacteria. *Appl. Environ. Microbiol.* **69**, 3036–3047 (2003).
19. Omand, M. M., Govindarajan, R., He, J. & Mahadevan, A. Sinking flux of particulate organic matter in the oceans: Sensitivity to particle characteristics. *Sci Rep* **10**, 5582 (2020).
20. Alldredge, A. The carbon, nitrogen and mass content of marine snow as a function of aggregate size. *Deep Sea Research Part I: Oceanographic Research Papers* **45**, 529–541 (1998).
21. McDonnell, A. M. P. & Buesseler, K. O. Variability in the average sinking velocity of marine particles. *Limnology and Oceanography* **55**, 2085–2096 (2010).
22. Jackson, G. A. Comparing observed changes in particle size spectra with those predicted using coagulation theory. *Deep Sea Research Part II: Topical Studies in Oceanography* **42**, 159–184 (1995).

23. Cram, J. A. *et al.* The Role of Particle Size, Ballast, Temperature, and Oxygen in the Sinking Flux to the Deep Sea. *Global Biogeochemical Cycles* **32**, 858–876 (2018).

24. Martin, J. H., Knauer, G. A., Karl, D. M. & Broenkow, W. W. VERTEX: carbon cycling in the northeast Pacific. *Deep Sea Research Part A. Oceanographic Research Papers* **34**, 267–285 (1987).

25. Cael, B. B. & Bisson, K. Particle Flux Parameterizations: Quantitative and Mechanistic Similarities and Differences. *Front. Mar. Sci.* **5**, (2018).

26. Iversen, M. H., Nowald, N., Ploug, H., Jackson, G. A. & Fischer, G. High resolution profiles of vertical particulate organic matter export off Cape Blanc, Mauritania: Degradation processes and ballasting effects. *Deep Sea Research Part I: Oceanographic Research Papers* **57**, 771–784 (2010).

27. Zakem, E. J. & Levine, N. M. Systematic Variation in Marine Dissolved Organic Matter Stoichiometry and Remineralization Ratios as a Function of Lability. *Global Biogeochem. Cycles* 2019GB006375 (2019) doi:10.1029/2019GB006375.

28. Guidi, L. *et al.* A new look at ocean carbon remineralization for estimating deepwater sequestration. *Global Biogeochemical Cycles* **29**, 1044–1059 (2015).

29. Palevsky, H. I. & Doney, S. C. How Choice of Depth Horizon Influences the Estimated Spatial Patterns and Global Magnitude of Ocean Carbon Export Flux. *Geophys. Res. Lett.* **45**, 4171–4179 (2018).

30. Zakem, E. J., Cael, B. B. & Levine, N. M. A unified theory for organic matter accumulation. *PNAS* (2021) doi:10.1101/2020.09.25.314021.

31. Forney, D. C. & Rothman, D. H. Common structure in the heterogeneity of plant-matter decay. *Journal of The Royal Society Interface* **9**, 2255–2267 (2012).

32. Lampitt, R. S., Wishner, K. F., Turley, C. M. & Angel, M. V. Marine snow studies in the Northeast Atlantic Ocean: distribution, composition and role as a food source for migrating plankton. *Marine Biology* **116**, 689–702 (1993).

33. Smith, D. C., Simon, M., Alldredge, A. L. & Azam, F. Intense hydrolytic enzyme activity on marine aggregates and implications for rapid particle dissolution. *Nature* **359**, 139–142 (1992).

34. Lauro, F. M. *et al.* The genomic basis of trophic strategy in marine bacteria. *PNAS* **106**, 15527–15533 (2009).

35. Levin, P. A. & Angert, E. R. Small but Mighty: Cell Size and Bacteria. *Cold Spring Harb Perspect Biol* **7**, (2015).

36. Lee, S. & Fuhrman, J. A. Relationships between Biovolume and Biomass of Naturally Derived Marine Bacterioplankton. *Appl. Environ. Microbiol.* **53**, 1298–1303 (1987).

37. Kaul, R. B., Kramer, A. M., Dobbs, F. C. & Drake, J. M. Experimental demonstration of an Allee effect in microbial populations. *Biology Letters* **12**, 20160070 (2016).

38. Boscolo-Galazzo, F. *et al.* Temperature controls carbon cycling and biological evolution in the ocean twilight zone. *Science* **371**, 1148–1152 (2021).

39. DeVries, T. & Weber, T. The export and fate of organic matter in the ocean: New constraints from combining satellite and oceanographic tracer observations. *Global Biogeochemical Cycles* **31**, 535–555 (2017).

40. Marsay, C. M. *et al.* Attenuation of sinking particulate organic carbon flux through the mesopelagic ocean. *PNAS* **112**, 1089–1094 (2015).

41. Dutkiewicz, S. *et al.* Capturing optically important constituents and properties in a marine biogeochemical and ecosystem model. *Biogeosciences* **12**, 4447–4481 (2015).

42. Savoie, E. R. *et al.* Ecophysiology of the Cosmopolitan OM252 Bacterioplankton (Gammaproteobacteria). **6**, 22 (2021).

43. Yung, C.-M. *et al.* Thermally adaptive tradeoffs in closely related marine bacterial strains. *Environmental Microbiology* **17**, 2421–2429 (2015).

44. Sannino, F. *et al.* A novel synthetic medium and expression system for subzero growth and recombinant protein production in Pseudoalteromonas haloplanktis TAC125. *Appl Microbiol Biotechnol* **101**, 725–734 (2017).

45. Aumont, O. *et al.* Variable reactivity of particulate organic matter in a global ocean biogeochemical model. *Biogeosciences* **14**, 2321–2341 (2017).

46. Boyd, P. W. *et al.* Transformations of biogenic particulates from the pelagic to the deep ocean realm. *Deep Sea Research Part II: Topical Studies in Oceanography* **46**, 2761–2792 (1999).

47. Schmidt, S., Chou, L. & Hall, I. R. Particle residence times in surface waters over the north-western Iberian Margin: comparison of pre-upwelling and winter periods. *Journal of Marine Systems* **32**, 3–11 (2002).

48. Arnosti, C. Patterns of Microbially Driven Carbon Cycling in the Ocean: Links between Extracellular Enzymes and Microbial Communities. *Advances in Oceanography* **2014**, e706082 (2014).

49. Berlemont, R. & Martiny, A. C. Glycoside Hydrolases across Environmental Microbial Communities. *PLOS Computational Biology* **12**, e1005300 (2016).

50. Kabisch, A. *et al.* Functional characterization of polysaccharide utilization loci in the marine Bacteroidetes 'Gramella forsetii' KT0803. *ISME J* **8**, 1492–1502 (2014).

51. Weiss, M. S. *et al.* Molecular architecture and electrostatic properties of a bacterial porin. *Science* **254**, 1627–1630 (1991).

52. Nguyen, T. T. H., Myrold, D. D. & Mueller, R. S. Distributions of Extracellular Peptidases Across Prokaryotic Genomes Reflect Phylogeny and Habitat. *Front. Microbiol.* **10**, (2019).

53. Arnosti, C. Microbial extracellular enzymes and the marine carbon cycle. *Ann Rev Mar Sci* **3**, 401–425 (2011).

54. Reintjes, G., Arnosti, C., Fuchs, B. & Amann, R. Selfish, sharing and scavenging bacteria in the Atlantic Ocean: a biogeographical study of bacterial substrate utilisation. *ISME J* **13**, 1119–1132 (2019).

55. Reintjes, G., Fuchs, B. M., Amann, R. & Arnosti, C. Extensive Microbial Processing of Polysaccharides in the South Pacific Gyre via Selfish Uptake and Extracellular Hydrolysis. *Front. Microbiol.* **0**, (2020).

56. Arnosti, C., Reintjes, G. & Amann, R. A mechanistic microbial underpinning for the size-reactivity continuum of dissolved organic carbon degradation. *Marine Chemistry* **206**, 93–99 (2018).

57. Cael, B. B. & White, A. E. Sinking Versus Suspended Particle Size Distributions in the North Pacific Subtropical Gyre. *Geophysical Research Letters* **47**, e2020GL087825 (2020).

58. Datta, M. S., Sliwerska, E., Gore, J., Polz, M. F. & Cordero, O. X. Microbial interactions lead to rapid micro-scale successions on model marine particles. *Nature Communications* **7**, 11965 (2016).

59. Amarnath, K. *et al. Stress-induced cross-feeding of internal metabolites provides a dynamic mechanism of microbial cooperation*. 2021.06.24.449802 https://www.biorxiv.org/content/10.1101/2021.06.24.449802v1 (2021) doi:10.1101/2021.06.24.449802.
