# Supplementary material for: Microbes contribute to setting the ocean carbon flux by altering the fate of sinking particulates
Source: Nat Commun. 2022 Mar 29;13:1657. doi: 10.1038/s41467-022-29297-2 (PMC8964765; doi:10.1038/s41467-022-29297-2)
Supplement: Supplementary file 3 — Reporting Summary [file 41467_2022_29297_MOESM3_ESM.pdf]

nature portfolio

Corresponding author(s): Naomi M Levine

Last updated by author(s): 2022/02/02

Reporting Summary

Nature Portfolio wishes to improve the reproducibility of the work that we publish. This form provides structure for consistency and transparency in reporting. For further information on Nature Portfolio policies, see our Editorial Policies and the Editorial Policy Checklist.

Statistics

For all statistical analyses, confirm that the following items are present in the figure legend, table legend, main text, or Methods section.

n/a	Confirmed	
☐	☒	The exact sample size (*n*) for each experimental group/condition, given as a discrete number and unit of measurement
☒	☐	A statement on whether measurements were taken from distinct samples or whether the same sample was measured repeatedly
☒	☐	The statistical test(s) used AND whether they are one- or two-sided<br>*Only common tests should be described solely by name; describe more complex techniques in the Methods section.*
☒	☐	A description of all covariates tested
☒	☐	A description of any assumptions or corrections, such as tests of normality and adjustment for multiple comparisons
☒	☐	A full description of the statistical parameters including central tendency (e.g. means) or other basic estimates (e.g. regression coefficient) AND variation (e.g. standard deviation) or associated estimates of uncertainty (e.g. confidence intervals)
☒	☐	For null hypothesis testing, the test statistic (e.g. *F*, *t*, *r*) with confidence intervals, effect sizes, degrees of freedom and *P* value noted<br>*Give P values as exact values whenever suitable.*
☒	☐	For Bayesian analysis, information on the choice of priors and Markov chain Monte Carlo settings
☒	☐	For hierarchical and complex designs, identification of the appropriate level for tests and full reporting of outcomes
☒	☐	Estimates of effect sizes (e.g. Cohen's *d*, Pearson's *r*), indicating how they were calculated

Our web collection on statistics for biologists contains articles on many of the points above.

Software and code

Policy information about availability of computer code

Data collection	The model code is deposited on GitHub (https://github.com/LevineLab/POMmodel.git) and citable using DOI: 10.5281/zenodo.6015020
Data analysis	No software or custom code was used.

For manuscripts utilizing custom algorithms or software that are central to the research but not yet described in published literature, software must be made available to editors and reviewers. We strongly encourage code deposition in a community repository (e.g. GitHub). See the Nature Portfolio guidelines for submitting code & software for further information.

Data

Policy information about availability of data

All manuscripts must include a data availability statement. This statement should provide the following information, where applicable:

- Accession codes, unique identifiers, or web links for publicly available datasets
- A description of any restrictions on data availability
- For clinical datasets or third party data, please ensure that the statement adheres to our policy

No new data is presented in the primary analysis for the paper. We include 12 new measurements of bacterial growth rates at different temperatures to support supplemental figure S5. These new data and collection method are fully provided in the Supplement (Table S2 and caption).

Field-specific reporting

Please select the one below that is the best fit for your research. If you are not sure, read the appropriate sections before making your selection.

☐ Life sciences ☐ Behavioural & social sciences ☒ Ecological, evolutionary & environmental sciences

For a reference copy of the document with all sections, see nature.com/documents/nr-reporting-summary-flat.pdf

Ecological, evolutionary & environmental sciences study design

All studies must disclose on these points even when the disclosure is negative.

Study description	New mathematical model of microbial growth on particles in the ocean
Research sample	No new samples were generated through the study. The model simulations were conducted to represent both published laboratory cultured experiments and global in situ samples The parameter values were selected to reflect this wide range.
Sampling strategy	The model parameters were stochastically assigned within the ranges defined based on the literature.
Data collection	The model was run for 600 days and the output was saved throughout the simulation.
Timing and spatial scale	See above description for sampling strategy and data collection
Data exclusions	No data was excluded
Reproducibility	Each type of stochastic model simulation was conducted 1000 times to ensure that parameter space was adequately sampled
Randomization	See above comments on sampling strategy and reproducibility
Blinding	Samples used to parameterize the models were not used for validation. Other types of blinding are not applicable to modeling studies.

Did the study involve field work? ☐ Yes ☒ No

Reporting for specific materials, systems and methods

We require information from authors about some types of materials, experimental systems and methods used in many studies. Here, indicate whether each material, system or method listed is relevant to your study. If you are not sure if a list item applies to your research, read the appropriate section before selecting a response.

Materials & experimental systems

n/a	Involved in the study	
☒	☐	Antibodies
☒	☐	Eukaryotic cell lines
☒	☐	Palaeontology and archaeology
☒	☐	Animals and other organisms
☒	☐	Human research participants
☒	☐	Clinical data
☒	☐	Dual use research of concern

Methods

n/a	Involved in the study	
☒	☐	ChIP-seq
☒	☐	Flow cytometry
☒	☐	MRI-based neuroimaging
